# Digital Mental Health Interventions for the Prevention and Treatment of Social Anxiety Disorder in Children, Adolescents, and Young Adults: Systematic Review and Meta-Analysis of Randomized Controlled Trials

**DOI:** 10.2196/67067

**Published:** 2025-06-12

**Authors:** Noemi Walder, Alessja Frey, Thomas Berger, Stefanie Julia Schmidt

**Affiliations:** 1 Division of Clinical Child and Adolescent Psychology Institute of Psychology University of Bern Bern Switzerland; 2 Division of Clinical Psychology and Psychotherapy Institute of Psychology University of Bern Bern Switzerland

**Keywords:** social anxiety, digital mental health intervention, digital mental health prevention, children, adolescents, young adults, meta-analysis, systematic review

## Abstract

**Background:**

Social anxiety disorder (SAD) substantially affects young individuals’ social and academic functioning, emphasizing the need for accessible and effective treatments such as digital mental health interventions (DMHIs).

**Objective:**

This systematic review and meta-analysis aimed to evaluate the efficacy of DMHIs for children, adolescents, and young adults with social anxiety symptoms.

**Methods:**

For this systematic review and meta-analysis, we searched 6 electronic databases (PsycINFO, Embase, MEDLINE, PSYNDEX, PubMed, and Web of Science) for randomized controlled trials investigating DMHIs addressing social anxiety in young people (mean age <25 years). Two authors independently screened the records, extracted data, and assessed the risk of bias. For data analysis, a standardized effect size was calculated using Hedges *g*, along with 95% CIs, for each study. Meta-analyses were conducted using a random-effects model to account for heterogeneity.

**Results:**

The systematic review included 22 studies, and the meta-analysis included 21 studies. The results significantly favored DMHIs (Hedges *g*=0.508, 95% CI 0.308-0.707; *P*<.001) over any control condition (ie, waitlist or active interventions) after the intervention, specifically those compared to waitlist control conditions (Hedges *g*=0.576, 95% CI 0.343-0.809; *P*<.001), those based on cognitive behavioral principles (Hedges *g*=0.610, 95% CI 0.361-0.859; *P*<.001), those incorporating SAD-specific components (Hedges *g*=0.878, 95% CI 0.469-1.278), and those delivered with human guidance (Hedges *g*=0.825, 95% CI 0.425-1.224; *P*<.001). Neither parental involvement nor age influenced outcomes significantly. When publication bias was considered, the overall effect remained significant (Hedges *g*=0.506, 95% CI 0.308-0.707). The risk-of-bias assessment indicated that most of the studies (16/22, 73%) showed some concerns; of the 22 studies, 3 (14%) were classified as high risk, and 3 (14%) were rated as low risk. The reporting of adherence varied substantially and could not be analyzed meta-analytically.

**Conclusions:**

The meta-analysis supports the efficacy of DMHIs for social anxiety compared to control conditions and the beneficial effects of guidance and interventions specifically designed for SAD. Furthermore, it highlights methodological shortcomings and heterogeneous reporting standards. Future research should prioritize higher methodological quality and should explore how effects are related to age and specific intervention components, including guidance and treatment modules.

**Trial Registration:**

PROSPERO CRD42023424181; https://www.crd.york.ac.uk/PROSPERO/view/CRD42023424181

## Introduction

### Social Anxiety Disorder in Children, Adolescents, and Young Adults

Anxiety disorders are the most prevalent mental disorders in children, adolescents, and young adults [1-3]. Especially in adolescence, social anxiety disorder (SAD) is particularly common, with prevalence rates ranging from 12% to 36% [4,5]. Notably, 88% of individuals with SAD experience onset before the age of 25 years and 50% before the age of 14 years [6]. The first symptoms (eg, shyness and fear of embarrassment) often appear in childhood [7,8]; and in adolescence, up to 50% of individuals report subclinical levels of social anxiety symptoms [9,10].

SAD is characterized by an intense and persistent fear of embarrassing oneself in front of others or being judged negatively by others [11]. To endure feared situations, those affected by SAD often engage in safety behaviors to minimize negative evaluations or avoid social situations altogether [11]. These behaviors maintain social anxiety symptoms [12-14]; contribute to substantial impairment in their psychosocial functioning (eg, fewer friends than their healthy peers) and the development of comorbid disorders (eg, depression and substance use); and lead to negative long-term effects, such as poorer academic and job opportunities and a lack of social support due to a small or nonexistent social network [15-20]. These detrimental consequences demand prevention efforts and effective treatments to intervene early.

### Efficacy of Treatment and Barriers to Treatment

Established treatment protocols (eg, those based on the cognitive model developed by Clark and Wells [12]) in adults have been adapted to suit the specific needs of children and adolescents [21]. Meta-analyses support their efficacy in individual and group settings compared to waitlist or psychological placebo control groups (ie, attention control) [22-24]. Accordingly, individual or group cognitive behavioral therapy (CBT) is recommended as a first-line treatment for children and adolescents with SAD [25,26].

Although effective treatments exist, treatment delivery and uptake are impeded by poor mental health literacy (eg, poor symptom recognition); public and self-stigma, particularly in individuals with SAD; and a gap in the provision of care [27-30]. The care gap is further exacerbated in children and adolescents, individuals from ethnic minority groups, those living in rural areas or lower-income countries, and those with lower socioeconomic status [31-33]. Various efforts have addressed some of these barriers by integrating care into primary physician settings, implementing psychoeducational interventions in educational settings, or using task-sharing approaches (eg, interventions delivered by nonprofessionals under supervision) [34-36]. Digital interventions are a highly scalable and far-reaching solution that may address barriers not addressed by previous efforts (ie, limitations related to time and location).

### Digital Mental Health Interventions for Social Anxiety

Digital mental health interventions (DMHIs) aim to manage, alleviate, or treat mental health problems through a digital medium (ie, internet, mobile phone app, wearables, or SMS text messaging) [37,38]. They can include some form of human support (ie, guided self-help), for instance, delivered through asynchronous messages, synchronous real-time chat, or telephone calls with a professional; or they can be stand-alone interventions (ie, unguided self-help) [39]. Several DMHIs have been developed for adults with SAD, with the evidence base for SAD being among the most extensive, including interventions based on CBT, psychodynamic therapy, interpersonal therapy, and acceptance-based CBT [39,40]. For this age group, meta-analytic evidence supports the efficacy of DMHIs: internet-delivered CBT (iCBT) and virtual reality exposure significantly outperformed passive or waitlist control conditions. Compared to other active control conditions, iCBT yielded small effects and was found to be as efficacious as face-to-face psychotherapy [41-43].

In children and adolescents, meta-analyses on the efficacy of DMHIs have focused predominantly on depressive and anxiety symptoms but have not reported effect sizes for either the prevention or the treatment of SAD specifically [37,38]. Although SAD outcomes were presented separately in 1 meta-analysis, no pooled effect size was calculated [44]. For DMHIs for anxiety disorders in general, meta-analyses reported significant effects for interventions based on CBT principles compared to a waitlist control condition (ages 6-16 years: standardized mean difference [SMD] 0.68; ages 7-18 years: Hedges *g*=1.41; and ages 6-25 years: Hedges *g=*0.68) and no significant difference when compared to other active conditions (eg, face-to-face therapy: Hedges *g*=0.30; SMD −0.04) [45-47]. For DMHIs for the prevention of anxiety disorders, meta-analyses did not yield significant overall effects, but some randomized controlled trials (RCTs) found small effects in favor of DMHIs [48,49]. Similarly, the meta-analyses of prevention programs for anxiety disorders in school-based settings reported small to medium effect sizes compared to passive control conditions after the intervention [50,51].

Notably, some studies suggest that young people with SAD benefit less from transdiagnostic interventions that target several anxiety disorders than young people with other anxiety disorders [52-54]. However, other studies found no difference between SAD-specific and transdiagnostic anxiety interventions for children and adolescents with SAD [55,56]. Thus, it would be relevant to compare the effects of DMHIs that specifically target SAD and those that target anxiety disorders in general. To the best of our knowledge, no systematic review and meta-analysis has investigated the effect of DMHIs on social anxiety in children and adolescents specifically.

### Moderators in Treatment Outcome

DMHIs are not equally effective for all children, adolescents, and young adults. In the subsequent section, we discuss the potential moderators of treatment efficacy, which include control conditions, the psychological principles of the interventions, age, adherence, and the extent of support. Generally, the effect sizes are larger when DMHIs are compared with inactive control conditions (eg, waitlist) than with active control conditions [57,58]. Most DMHIs are based on CBT principles [37], although when compared to DMHIs based on other psychological interventions (eg, cognitive bias modification [CBM] and mindfulness), they do not result in better effects [59]. Evidence concerning age shows that older adolescents and young adults generally benefit more from DMHIs than children [46,60,61]. Similarly, adherence is supposed to be linked to symptom improvement, reflecting compliance with predefined and recommended use and engagement with the intervention [62-64]. However, the results regarding the association between adherence and outcome are more complex. Some studies point to a direct link between adherence and symptom improvement [65] and some to an inverse relationship [66], while others identify only single aspects of adherence (eg, module completion) to be relevant for symptom improvement [67]. Furthermore, studies indicate that adherence itself is, in turn, influenced by symptom severity, which may thereby affect symptom reduction [62]. Overall, adherence plays an important role in the implementation of DMHIs, and human support is a known factor in improving adherence [68,69]. This support can be provided in the form of guidance by professionals (ie, psychologists and therapists) or through the social environment of the young people (ie, parents and teachers).

### Objectives of This Systematic Review and Meta-Analysis

DMHIs are especially promising in young people because they are digital natives and generally seek help later and to a lesser extent than adults [38,70,71]. Thus, the low-threshold and confidential nature of DMHIs may appeal especially to young people with SAD [30,72]. Accordingly, this systematic review and meta-analysis aims to evaluate the efficacy of DMHIs in reducing social anxiety symptoms in children, adolescents, and young adults.

Our primary research question is as follows:

- Are DMHIs efficacious in reducing social anxiety symptoms in children, adolescents, and young adults?

The secondary research question includes moderator analyses:

- Is the between-group effect size of DMHIs moderated by control condition, age, the underlying psychological principle of the DMHI (ie, CBT and other interventions), the intervention focus (SAD-specific or anxiety disorders in general), or human support (ie, guided self-help vs unguided self-help and parental involvement vs no involvement)?

In addition to evaluating efficacy, we will systematically synthesize and assess adherence to the DMHIs, focusing on how adherence is defined, measured, and reported across studies.

## Methods

The protocol for this systematic review and meta-analysis was registered with PROSPERO (CRD42023424181) on August 15, 2023. The systematic review and meta-analysis were conducted in accordance with the PRISMA (Preferred Reporting Items for Systematic Reviews and Meta-Analyses) guidelines (Multimedia Appendix 1).

### Search Strategy and Study Selection

#### Search Strategy

We searched 6 electronic databases (PsycINFO, Embase, MEDLINE, PSYNDEX, PubMed, and Web of Science) on July 10, 2024. The search was restricted to journal articles in the PsycINFO and MEDLINE databases and RCTs in the Embase, MEDLINE, and PubMed databases. The search strings used for the systematic literature review are provided in Multimedia Appendix 2. Furthermore, the clinical trials database ClinicalTrials.gov was searched on July 31, 2024, for studies not identified in the electronic database search, which yielded no additional studies. Previously published meta-analyses on DMHIs for anxiety disorders in children and adolescents were checked for studies not identified in the database search.

#### Eligibility Criteria

Studies were included if they met the following inclusion criteria: (1) included young people with a mean age between 0 and <25 years [73,74]; (2) evaluated a DMHI based on psychological principles that could be delivered remotely; (3) used an RCT design comparing an experimental group to another active intervention, a waitlist, or a care-as-usual or treatment-as-usual condition; (4) reported an outcome measure assessing social anxiety; and (5) were published in English or German in a peer-reviewed journal. Study protocols, reviews, and meta-analyses were excluded.

The age range of 0 to <25 years was selected to reflect current theories in child and adolescent psychiatry [75,76] and epidemiological evidence indicating that the onset of SAD often occurs within this age range [6]. The rationale for including both prevention and treatment across the diagnostic spectrum is based on the understanding that social anxiety symptoms exist on a continuum and that the intervention techniques underlying the prevention and treatment of SAD are similar [12,13,21,77-79].

#### Study Selection

Titles, abstracts, and full texts were systematically reviewed. Author NW extracted all relevant information from titles and abstracts into Excel (Microsoft Corp) for the study selection process. Independently, authors NW and AF first screened all titles and then all abstracts of the records identified through the searches. No specialized software was used. In cases of discrepancies between the raters, the records were advanced to the next screening round. Afterward, NW and AF reviewed the full texts of the remaining records. If disagreements arose regarding inclusion or exclusion, they discussed each decision until reaching consensus. If no agreement could be reached, the final decision was taken together with author SJS. As a consensus-based method was followed, interrater reliability was not calculated.

### Data Extraction

Two authors (NW and AF) independently extracted the data into a preformatted Excel file based on the Cochrane data collection form from the study by Higgins et al [80]. No automation tools were used. Information extracted from primary studies included the following: study characteristics (ie, country, ethics approval, trial preregistration, funding, study aims, study design, recruitment method, randomization procedure, justification of sample size, dropouts, and handling of missing values), sample characteristics (ie, informed consent procedure, eligibility criteria, sample size, sociodemographic information, and clinical status), intervention characteristics (description of the intervention; underlying psychological principles; duration, number of modules, and delivery setting of the intervention; extent of guidance; guidance provider and their training; and adherence to and satisfaction with the intervention), control condition characteristics (ie, waitlist; care as usual; treatment as usual; or another active intervention, including description and underlying psychological principles), and outcome data for all available measurement time points (ie, assessment tool, sample size, unstandardized means, SDs, and SEs if SDs were not available). If transdiagnostic anxiety measures were used, and no outcome data for SAD were reported, the corresponding authors were contacted.

### Data Analyses

Data analyses followed a practical guide for meta-analyses in mental health research [81] and were analyzed with Comprehensive Meta-Analysis software (version 4.0; Biostat, Inc) [82]. For each study, a standardized effect size was computed using Hedges *g* and 95% CIs derived from means, SDs, and sample sizes of the intervention and control groups. To account for potential heterogeneity, all analyses were computed with random-effects models [81]. In 3-armed RCTs, we prioritized comparisons with the waitlist control group over another active control group because there were more studies with passive comparison conditions, leading to a more homogeneous comparison group [83-86].

Heterogeneity was assessed through visual inspection of the forest plot, the Cochran Q test, and the calculation of the *I*^2^ statistic. The Q test assesses whether the observed effect sizes significantly differ from each other beyond chance. A significant Q statistic indicates heterogeneity, but in small meta-analyses, there might be insufficient power to detect it [81]. *I*^2^ values explain the proportion of heterogeneity, that is, the variability in treatment effect estimates due to real study differences and not due to chance, with thresholds of 25%, 50%, and 75% indicating low, moderate, and high heterogeneity, respectively [87]. In addition, we calculated tau and tau squared. Tau measures the SD of the true effects across studies, while tau squared is its square representing the estimated between-study variance. A significant tau-squared value suggests heterogeneity, supporting the use of random-effects models [88].

As high heterogeneity was expected, we explored possible sources of heterogeneity in moderator analyses. Moderators were explored in subgroup analyses if the moderator was categorical (ie, control condition, risk of bias, psychological principles, SAD-specific intervention, guidance, parental involvement, or age group) or in a meta-regression analysis if the moderator was continuous (ie, age or symptom severity). Subgroup analyses included the computation of average effect sizes and the Q statistic as an indicator of a common effect size. A significant Q statistic suggests heterogeneity in effect sizes within the subgroup.

Sensitivity analyses were performed to investigate the robustness of our findings. We conducted analyses excluding studies with the highest and lowest effect sizes to assess the potential influence of outliers on the meta-analytic results. This allowed us to evaluate the stability of the overall effect size when extreme values were omitted. In another sensitivity analysis, we only included studies in which all participants met diagnostic criteria for SAD to control for heterogeneous diagnostic samples. Moreover, in a subgroup analysis, studies were stratified based on their risk-of-bias ratings to investigate the influence of the methodological quality. In addition to these preregistered analyses, we conducted a sensitivity analysis by excluding studies with participants aged >25 years to test whether older participants altered the effects, and we carried out a moderator analysis with age groups such as children (<11 years), adolescents (11-17 years), and young adults (18-25 years) as moderators.

In addition, numbers needed to treat were computed for all effect sizes using the *dmetar* package in R with the default Kraemer and Kupfer method [89]. Numbers needed to treat indicate the number of participants to be treated to achieve 1 case with reliable symptom change.

To address a possible publication bias, we visually inspected the funnel plot for symmetry and conducted a Begg and Mazumdar rank correlation test deter and an Egger linear regression test to quantify asymmetry. Using the trim-and-fill approach proposed by Duval and Tweedie [90], we assessed missing studies in the funnel plot to estimate the effect size after imputation [81,91,92].

### Risk-of-Bias Assessment

Risk of bias was assessed by 2 authors (NW and AF) independently following the revised Cochrane Risk of Bias Tool for Randomized Trials (RoB 2) procedure and using the RoB 2 Excel template [93]. After the individual rating, discrepancies between ratings were discussed until consensus was reached. The RoB 2 tool includes five domains for randomized trials: (1) bias arising from the randomization process, (2) bias due to deviations from intended interventions, (3) bias due to missing outcome data, (4) bias in measurement of the outcome, and (5) bias in selection of the reported result. Risk of bias was rated separately for each domain based on whether it was likely to have influenced study outcomes. Studies categorized as low overall risk of bias were rated as low risk of bias across all 5 domains. A rating of “some concerns” in at least 1 domain led to an overall judgment of “some concerns” for the study. Studies were rated as high risk of bias if at least 1 domain was rated as high risk [81].

## Results

### Study Selection

**Figure 1:**
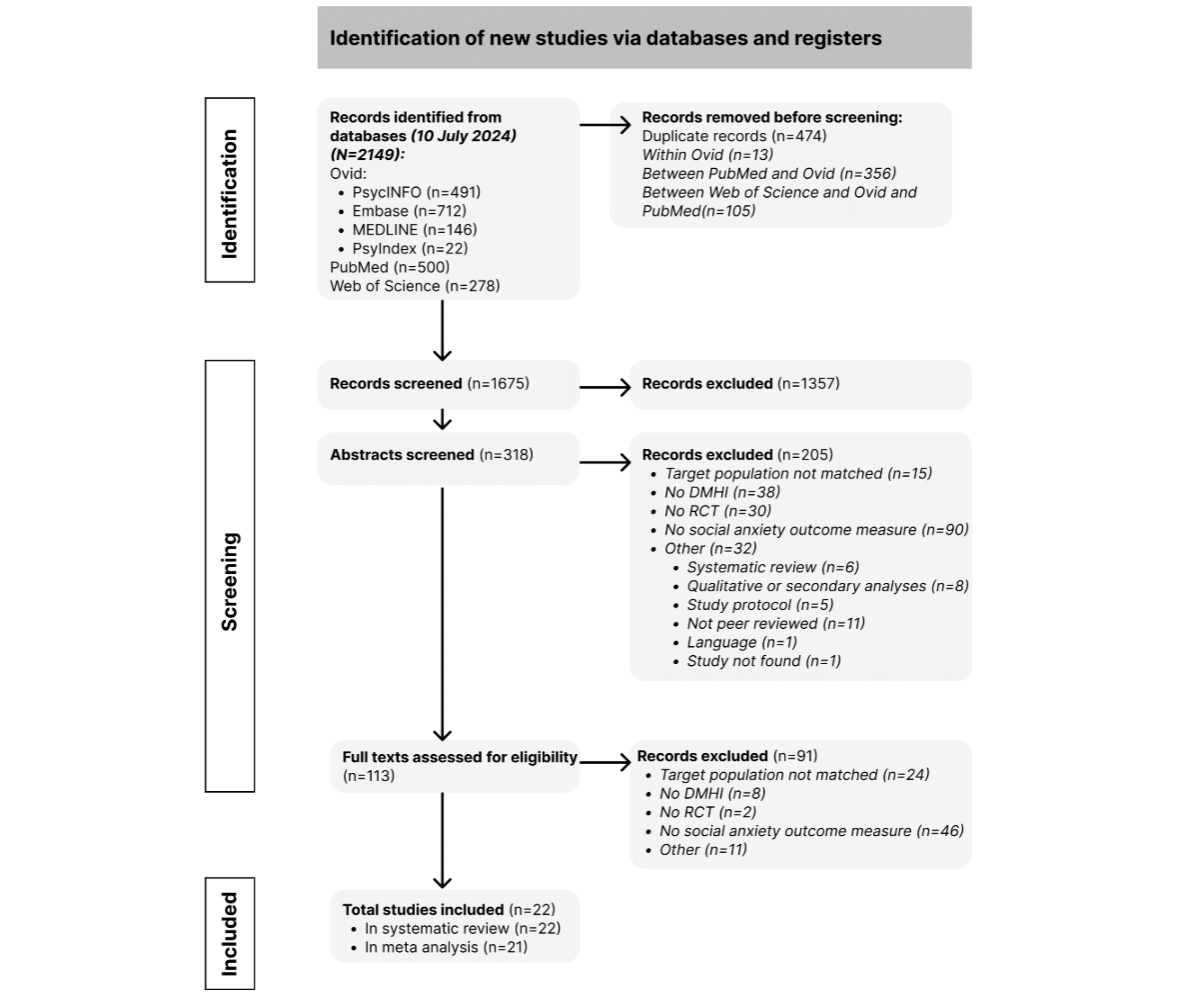
PRISMA (Preferred Reporting Items for Systematic Reviews and Meta-Analyses) flow diagram, depicting the study identification and screening process. DMHI: digital mental health intervention; RCT: randomized controlled trial.

The systematic literature search yielded 2149 records across all electronic databases, from which 474 (22.06%) duplicates were removed before screening. Of the remaining 1675 records, due to nonfulfillment of eligibility criteria, 1357 (81.01%) were excluded after title screening, and 205 (12.23%) were removed after abstract screening. Therefore, 113 full texts were assessed for eligibility, of which 22 (19.5%) were ultimately included in the systematic review and 21 (18.6%) in the meta-analysis. The study by Vigerland et al [94] was not included in the meta-analytic calculations because there were insufficient data related to social anxiety at the postintervention assessment. A complete overview of the study selection process is presented in Figure 1.

### Study Characteristics

The included papers were published between 2011 and 2024. The 22 trials were conducted in Australia (n=7, 32%), the United States (n=4, 18%), Sweden (n=3, 14%), the United Kingdom (n=2, 9%), China (n=2, 9%), Spain (n=1, 5%), Iran (n=1, 5%), Canada (n=1, 5%), and the Netherlands (n=1, 5%). A detailed overview of study characteristics is presented in Tables 1 and 2.

All non–US currency values were converted to US dollars using exchange rates corresponding to the date of first manuscript submission for each study. Converted amounts are shown in parentheses.

**Table 1:** Overview of the included studies and participant characteristics (n=22).

Study; country	Participant characteristics						
	Recruitment strategy	N (intervention group:control group)	Age (y), mean (SD; range)	Gender (%)	Diagnosed with SAD^a^ (%)	Race and ethnicity (%)	Socioeconomic information
Soleimani Rad et al [95], 2024; Iran	School-based screening	54 (18:18:18)	17 (1.16; 15-19)	Female: 100	100	Iranian: 100	Average economic status: 84.3%; lower economic status: 15.5%
Espinosa et al [96], 2024; Spain	Self-selected sample: referrals by school counsellors	58 (NR^b^)	14.9 (2.0; 12-18)	Female: 81; male: 19	31	White: 90	Yearly family income: >€10,000 (US $10,597): 23.2%; €10,000-€25,000 (US $10 597-$26,492): 57.1%; >€25,000 (US $26,492): 19.6%
Mao et al [97], 2023; China	School-based screening	30 (15:15)	15.1 (2.6; 14-17)	Female: 64.3; male: 35.7	No clinician assessed diagnosis, all scored in top 10% of SAS-A^c^	NR	NR
Hilt et al [98], 2023; United States	Self-selected sample: letters to guardians of 6th-9th grade students at public schools; word of mouth; study posters in public spaces; web-based advertisements	152 (80:72)	13.7 (0.9; 12-15)	Female: 58.6; male: 41.5	No clinician assessed diagnosis	American Indian: 0.7; Asian: 2; Black: 3.3; Hispanic: 10.5; non-Hispanic: 89.5; White: 82.3; multiracial: 10.5; other: 1.3	Median household income: US $90,000-100,000; recipients of government-assisted food program: 9.21%
Mueller and Cougle [99], 2023; United States	Convenience sample: web-based advertisements; student research pool	55 (28:27)	19.45 (2.1; NR)	Female: 85.2; male: 14.8	100	Asian: 5.5; Black: 12.1; Latinx: 22.2; non-Hispanic: 75.9; White: 77.7	NR
Leigh and Clark [100], 2022; United Kingdom	School-based screening	43 (22:21)	16.2 (1.1; 14-18)	Female: 91; male: 9	100	Ethnic minority backgrounds: 48.8	NR
Bautista et al [101], 2022; United States	Self-selected sample: flyers on university campus	35 (20:15)	21.9 (4.8; 19-24)	Female: 71.4; male: 28.6	No clinician assessed diagnosis	African American: 11.4; Asian or Asian American or Pacific Islander: 17.1; European American: 71.4; Hispanic or Latinx: 8.6	NR
Schniering et al [102], 2022; Australia	Clinical sample: adolescents and their parents contacting the clinic for treatment were initially screened	91 (45:46)	14.3 (1.6; 12-17)	Female: 66; male: 34	28.5	NR	NR
Nordh et al [103], 2021; Sweden	Self-selected sample: referrals from health care professionals; advertisements at CAMHS^d^ clinics, in newspapers, and on social media	103 (51:52)	14.1 (2.1; 10-17)	Female: 77; male: 23.0	100	NR	NR
Stapinski et al [104], 2021; Australia	Self-selected sample: media coverage; social media postings	123 (62:61)	21.6 (2.2; 17-24)	Female: 67; male: 31.7; diverse: 0.8	No clinician assessed diagnosis	Australian-born: 82.1; of other origin: 17.9	NR
Wang et al [86], 2020; China	Self-selected sample: advertisements on different internet platforms	Randomized: 210 (70:70:70); analyzed: 104 (33:37:24)	24.9 (4.6; 18-45)	Female: 70.2; male: 29.8	100	NR	NR
Farrer et al [105], 2019; Australia	Self-selected sample: social media postings; school-based through newspaper; email	200	22.1 (4.9; 18<)	Female: 77.5; male: 17.0; diverse: 5.5	No clinician assessed diagnosis	Aboriginal or Torres Strait Islander or Pacific Islander: 1; African: 1; Asian or Indian: 28; Latinx or South American: 1.5; White or European: 64.5; other: 4	No financial stress: 28%; occasional financial stress: 42.5%; frequent financial stress: 21%; constant financial stress: 8.5%
McCall et al [106], 2018; Canada	Convenience sample: student research pool	101 (51:50)	21.9 (5.5; 17-46)	Female: 72; male: 28	No clinician assessed diagnosis, elevated score on Mini-SPIN and no prior SAD diagnosis	Asian: 62; White: 18; other: 20	NR
Spence et al [56], 2017, and Hearn et al [107], 2018)^e^; Australia	Self-selected sample: via schools, parent groups, mental health professionals, guidance officers, media, and Facebook	125; (47:48:30)	11.3 (2.7; 8-17)	Female: 60; male: 40	100	Place of birth—Australia: 83.2; United States or Canada: 4.8; United Kingdom: 4; New Zealand: 2.4; Europe: 1.6; Africa: 1.6; Asia: 0.8; other: 1.6; Indigenous Australian: 2.4	High income (≥Aus $100,001 (US $73,941): 57.9%; low and middle income (≤100,000; US $73,940): 42%
Morgan et al [108], 2017; Australia	Self-selected sample: paid Google and Facebook advertisements; postings on parenting and mental health websites; flyers in preschool services	433 (215:218)	4.8 (1.0; 3-6)	Female: 52.3; male: 47.7	No clinician assessed diagnosis	NR	Financial difficulty: 17.1%
Sanchez et al [109], 2017; United States	Self-selected sample: social media postings; postings on parenting listserves; referrals by national school systems and child service providers	69 (33:36)	8.9 (1.2; 7-11)	Female: 40.5; male: 59.5	No clinician assessed diagnosis	Hispanic and non-Hispanic—intervention group: 6 and 94, respectively; control group: 6 and 94, respectively; White and racial and ethnic minority people—intervention group: 55 and 45, respectively; control group: 56 and 44, respectively	NR
Calear et al [110], 2016; Australia	School-based sample: recruited schools from the Australian Capital Territory and South Australia	225 (127:98)	15.0 (1.08; 13-17)	Female: 70.6; male: 16.8	No clinician assessed diagnosis	NR	NR
Calear et al [84], 2016; Australia	School-based sample of 6 participating Headspace centers	1767 (NR)	14.8 (1.0; 12-18)	Female: 62.8; male: 37.2	No clinician assessed diagnosis	NR	NR
Vigerland et al [94], 2016; Sweden	Self-selected sample: media advertisements	93 (46:47)	10.1 (1.7; 8-12)	Female: 55; male: 45	10	NR	NR
Sportel et al [85], 2013; Netherlands	N/A^f^	240 (84:86:70)	14.1 (0.6; 12-15)	Female: 73.3; male: 26.7	Met SAD diagnosis: 12.9%; remaining had elevated symptoms of social and test anxiety	NR	NR
Bowler et al [83], 2012; United Kingdom	Self-selected sample: postpose; email campaign	71 (24:25:22)	22.7 (5.9; 18-48)	Female: n=43 (68.3); male: n=20 (31.7)	No clinician assessed diagnosis; elevated SPIN^g^ scores	Primarily White	NR
Tillfors et al [111], 2011; Sweden	Self-selected sample: regional newspaper articular; school staff; advertisements in high school	19 (10:9)	16.5 (1.6; 15-21)	Female: 89; male: 11	100	NR	NR

^a^SAD: social anxiety disorder.

^b^NR: not reported.

^c^SAS-A: Social Anxiety Scale for Adolescents [112].

^d^CAMHS: Child and Adolescent Mental Health Services.

^e^The data reported by Spence et al [56] were later used by Hearn et al [107]. Spence et al [56] compared 3 arms of the RCT (transdiagnostic iCBT, SAD-specific iCBT, and waitlist control), while Hearn et al [107] compared both intervention groups to the waitlist group. Depending on the analysis, data were either drawn from Spence et al [56] or Hearn et al [107].

^f^N/A: not applicable.

^g^SPIN: Social Phobia Inventory (Connor et al [113]).

**Table 2:** Overview of the included studies, study characteristics, and dropout details (n=22).

Study; country	Study characteristics							Dropout (those without postintervention assessment), %
	Study design	Measurement time points	SAD^a^ outcome measure	Intervention used	Control condition	Control condition		
Soleimani Rad et al [95], 2024; Iran	RCT^b^ (3 arms)	Baseline to postintervention assessment: 14 wk; follow-up: 3 mo	SPIN^c^	iCBT^d^	Face-to-face CBT	Waitlist	24.3	
Espinosa et al [96], 2024; Spain	RCT (2 arms)	Baseline to postintervention assessment: 8 weeks; follow-up: 3 mo	RCADS^e^-30 social phobia subscale	AMtE^f^	UP-A^g^ telehealth administered	N/A^h^	16.7	
Mao et al [97], 2023; China	RCT (2 arms)	Baseline to postintervention assessment: 4 wk	SAS-A^i^	CBM-I^j^ app	Waitlist	N/A	6.6	
Hilt et al [98], 2023^k^; United States	RCT (2-arms)	Baseline to postintervention assessment: 3 wk; follow-up: 6 wk, 12 wk, 6 mo	MASC-SA^l^	App-based mindfulness exercises	Mood monitoring	N/A	0	
Mueller and Cougle [99], 2023; United States	RCT (2 arms)	Baseline to postintervention assessment: 4 wk; follow-up: 1 mo	SPIN	Building Closer Friendships	Waitlist	N/A	23.64	
Leigh and Clark [100], 2022; United Kingdom	RCT (2 arms)	Baseline to postintervention assessment: 14 wk; follow-up: 3 mo, 6 mo	LSASCA-SR^m^	OSCA^n^	Waitlist	N/A	4.65	
Bautista et al [101], 2022; United States	RCT (2 arms)	Baseline to postintervention assessment: 6 wk	SIAS^o^	Social phobia course at This Way Up Clinic	Waitlist	N/A	17.14	
Schniering et al [102], 2022^k^; Australia	RCT (2 arms)	Baseline to postintervention assessment: 8 wk; follow-up: 3 mo (intervention group only)	SCASy^p^	Chilled Plus Program	Waitlist	N/A	14.46	
Nordh et al [103], 2021; Sweden	RCT (2 arms)	Baseline to postintervention assessment: 10 wk; follow-up: 3 mo	LSAS-C^q^	iCBT for SAD	ISUPPORT^r^: psychoeducation about SAD and healthy habits	N/A	1.94	
Stapinski et al [104], 2021; Australia	RCT (2 arms)	Baseline to postintervention assessment: 2 mo; follow-up: 6 mo	Composite score of SIAS and SPIN	Inroads	Psychoeducation on alcohol	N/A	30.65	
Wang et al [86], 2020; China	RCT (3 arms)	Baseline to postintervention assessment: 8 wk	SIAS	Guided iCBT for SAD	Self-help iCBT for SAD	Waitlist	50.48	
Farrer et al [105], 2019; Australia	RCT (2 arms)	Baseline to postintervention assessment: 6 wk; follow-up: 3 mo	SOPHS^s^	Uni Virtual Clinic	Waitlist	N/A	28	
McCall et al [106], 2018; Canada	RCT (2 arms)	Baseline to postintervention assessment: 4 mo	SIAS	Overcome Social Anxiety	Waitlist	N/A	35.64	
Spence et al [56], 2017, and Hearn et al [107], 2018)^v^; Australia	RCT (3 arms)	Baseline to postintervention assessment: 12 wk; follow-up: 6 mo	SPAI-C^t^	iCBT (SAD)	iCBT (generic)	Waitlist	21.6	
Morgan et al [108], 2017; Australia	RCT (2 arms)	Baseline to postintervention assessment: 12 wk; follow-up: 24 wk	PAS-R^u^, SAD subscale	Cool Little Kids parenting group program	Waitlist	N/A	20.79	
Sanchez et al [109], 2017; United States	RCT (2 arms)	Baseline to postintervention assessment: 9 wk	SASC-R^v^	Adventures Aboard the S.S. Grin	Waitlist	N/A	NR	
Calear et al [110], 2016; Australia	Cluster RCT (2 arms)	Baseline to postintervention assessment: 6 wk; follow-up: 3 mo	SAS-A	e-Couch Anxiety and Worry program	Waitlist	N/A	Intervention group: 38.8; control group: 27:6	
Calear et al [84], 2016; Australia	Cluster RCT (3 arms)	Baseline to postintervention assessment: 6 wk; follow-up: 6 mo, 12 mo	SAS-A	e-GAD health service method	e-GAD school method	Waitlist	52.52	
Vigerland et al [94], 2016; Sweden	RCT (2 arms)	Baseline to postintervention assessment: 11 wk; follow-up: 3 mo; follow-up: 12 mo^w^	SPAI-C and SPAI-P^x^	iCBT	Waitlist	N/A	96.77	
Sportel et al [85], 2013; Netherlands	Cluster RCT (3 arms)	Baseline to postintervention assessment: 12 wk; follow-up: 6 mo, 12 mo	RCADS, SAD subscale	CBM-I	Group CBT	Waitlist	16.67	
Bowler et al [83], 2012; United Kingdom	RCT (3 arms)	Baseline to postintervention assessment: 2 wk	SPIN	e-Couch social anxiety program	CBM-I	Waitlist	11.27	
Tillfors et al [111], 2011; Sweden	RCT (2 arms)	Baseline to postintervention assessment: 9 wk; follow-up: 1 y	SPSQ-C^y^ and LSAS-SR^z^	CBT-based web-based intervention	Waitlist	N/A	5.26	

^a^SAD: social anxiety disorder.

^b^RCT: randomized controlled trial.

^c^SPIN: Social Phobia Inventory (Connor et al [113]).

^d^iCBT: internet-delivered cognitive behavioral therapy.

^e^RCADS: Revised Children’s Anxiety and Depression Scale [114].

^f^AMtE: Aprende a Manejar tus Emociones (Learn to Manage your Emotions)

^g^UP-A: Unified Protocol for Transdiagnostic Treatment of Emotional Disorders in Adolescents.

^h^N/A: not applicable.

^i^SAS-A: Social Anxiety Scale for Adolescents [112].

^j^CBM-I: cognitive bias modification for interpretation.

^k^We obtained unpublished, SAD-specific outcome data, which were not included in the original outcome paper, directly from the authors.

^l^MASC-SA: Multidimensional Anxiety Scale for Children, social anxiety subscale [115].

^m^LSASCA-SR: Liebowitz Social Anxiety Scale for Children and Adolescents, self-report version [116].

^n^OSCA: Online Social Anxiety Cognitive Therapy for Adolescents.

^o^SIAS: Social Interaction Anxiety Scale [117].

^p^SCASy: Spence Children's Anxiety Scale, youth report [118].

^q^LSAS-C: Liebowitz Social Anxiety Scale, child version [116].

^r^ISUPPORT: internet-delivered supportive therapy.

^s^SOPHS: Social Phobia Screener [119].

^t^SPAI-C: Social Phobia and Anxiety Inventory for Children [120].

^u^PAS-R: Preschool Anxiety Scale, revised [121].

^v^SASC-R: Social Anxiety Scale for Children, revised [122].

^w^12-month follow-up data were reported in a sperate publication [94].

^x^SPAI-P: Social Phobia and Anxiety Inventory for Children completed by the parent rather than the child [120].

^y^SPSQ-C: Social Phobia Screening Questionnaire for Children and Adolescents [123].

^z^LSAS-SR: Liebowitz Social Anxiety Scale–Self-Report [116].

### Sample Characteristics

Participants were recruited through various strategies, primarily using self-selected sampling procedures such as posters and flyers, word of mouth, social media and web-based advertisements, postings on platforms or forums used by parents and health care providers, and media and newsletter articles (13/22, 59%) [56,83,86,94,96,98,103-105,107-109,111]. The remaining RCTs (9/22, 41%) recruited participants via convenience sampling (ie, student research pool [99,106], school-based screening procedures [84,95,97,100,110], or an associated mental health clinic [102]). Only a few of the studies (2/22, 9%) mentioned efforts to ensure socioeconomic diversity, either by choosing schools representing a range of socioeconomic backgrounds [100] or by aligning the study cohort’s demographic profile with that of the general population [56].

In total, 4196 participants were randomly assigned across all RCTs. They ranged in age from 3 to 48 years, with mean ages spanning from 4.8 to 24.9 years. In all studies, more than half of the participants were female. A comprehensive compilation of participant-related characteristics, including ethnic diversity and socioeconomic information for the final samples, is presented in Tables 1 and 2.

### Intervention Characteristics

#### Overview

A detailed overview of intervention characteristics is presented in Table 3. Of the 22 studies, 20 (91%) delivered the intervention via computer, while 2 (9%) investigated a smartphone app [97,98]. In most of the studies (20/22, 91%), participants completed the interventions from home (or from any location). Only some of the studies (2/22, 9%) delivered the intervention in a laboratory [83] or school setting [84] under the observation of a researcher or a teacher, respectively. Of the 22 studies, 2 (9%) involved apps, 1 (5%) featured a social skills game, and 19 (86%) involved module-based interventions. Participants either had access to all modules from the beginning, or 1 module was activated per week.

**Table 3:** Characteristics of interventions.

Study	Psychological principle	SAD^a^ specific	Modules	Intervention duration	Adherence	Guidance format	Amount of guidance	Parental involvement
Soleimani Rad et al [95], 2024	Integrated CBT^b^-based program based on CBT manual for children with anxiety (Kendal and Hedtke [124]) and CBT for SAD (Hofmann and Otto [125])	Yes	10	10 wk	All 10 modules completed: 83.3%	Weekly 5-min video call by a licensed therapist	NR^c^	No
Espinosa et al [96], 2024	Transdiagnostic CBT program to support emotion regulation based on UP-A^d^ and previous Spanish program for adults	No	8	8 wk	≤1 modules completed: 100%; mean number of completed modules: 7.05 (SD 1.25)	One telephone call with adolescents and parents at the beginning and after intervention completion	NR	No
Mao et al [97], 2023	CBM^e^, including interpretation bias modification tasks	Yes	8	4 wk	Completed entire training: 93.3%	No guidance provided	No guidance provided	No
Hilt et al [98], 2023	Mood monitoring and mindfulness exercises	No	Patients received mindfulness exercises after mood monitoring question; exercise length depended on availability of patients: 1, 5, or 10 min	3 wk	NR	No guidance provided	No guidance provided	No
Mueller and Cougle [99], 2023	The Building Closer Friendships program consisted of three components: (1) emotional writing, (2) social skills, and (3) exposure exercises addressing individuals with SAD, specifically fear of intimacy	Yes	3 (including 10 treatment components)	4 wk	≤1 treatment component completed: 100%; mean number of completed treatment components: 7.65 (SD 2.38)	No guidance provided	No guidance provided	No
Leigh and Clark [100], 2022	OSCA^f^: CBT-based program for SAD based on the cognitive model developed by Clark and Wells [12]	Yes	8 core modules for weeks 1 and 2; up to 16 additional modules to individualize treatment on specific problems and fears	14 wk	Mean time spent on OSCA: 26.14 (SD 11.32) h; mean number of completed exercises: 25 (SD 10.75)	Weekly 20-min telephone calls and messages in the program by a clinical psychologist	Mean time spent in direct communication with participants: 398.67 (SD 59.38) min	No
Bautista et al [101], 2022	CBT-based program addressing SAD provided via This Way Up Clinic	Yes	6	6-10 wk	NR	Weekly in-person meetings by undergraduate students who participated in 3 training sessions	Mean meeting time: 11.73 (SD 6.57) min	No
Schniering et al [102], 2022	Chilled Plus is a CBT-based program addressing anxiety and depression based on the face-to-face Chilled program	No	8	8 wk	NR	Eight 30-min telephone calls by therapists	NR	Yes
Nordh et al [103], 2021	CBT-based program addressing SAD based on the program by Nordh et al [126]	Yes	10 for child and 5 for parent	10 wk	Mean patient adherence scale rating: 22.84 (SD 10.10)	Three 30-min telephone calls; asynchronous support by clinical psychologists	Mean time spent by therapists per wk: 28.85 (SD 16.79) min	Yes
Stapinski et al [104], 2021	Inroads: CBT-based program addressing anxiety and hazardous alcohol use	No	5	8 wk	≤1 module completed: 77%; ≤3 modules completed: 51%; all 5 modules completed: 39%	Weekly emails; two 30-min telephone calls or chat sessions by clinical psychologists	NR	No
Wang et al [86], 2020	CBT-based treatment addressing SAD based on the program developed by Berger et al [127] (guided and unguided self-help)	Yes	5	8 wk	Guided: NR; unguided self-help: NR	Weekly emails by psychology graduate students with training in CBT under supervision of a clinical psychologist; self-help: no guidance provided	Mean time spent for guidance per participant per wk: 15 min; self-help: no guidance provided	Guided: no; unguided self-help: no
Farrer et al [105], 2019	Uni Virtual Clinic: CBT-based web-based mental health program	No	Multiple modules covering topics that students might be affected by (eg, mood, anxiety, substance use, eating disorders and loneliness, homesickness, and adjusting to university)	6 wk	Accessed the program: 75.8%; logged in weekly: 63.8%; spent <5 min per visit: 42.6%	No guidance provided	No guidance provided	No
McCall et al [106], 2018	CBT-based Overcome Social Anxiety program addressing SAD	Yes	7	4-6 mo	NR	No guidance provided (automated emails)	No guidance provided	No
Spence et al [56], 2017; Hearn et al [107], 2018)	CBT-based generic anxiety program (BRAVE-ONLINE); CBT-based program addressing SAD that specifically focused on social anxiety and included social skills training and SAD-specific cognitive elements based on the cognitive model developed by Clark and Wells [12]	No; yes	10 (60 min each)	12 wk	Mean number of modules completed at postintervention assessment—children: 4.75 of 10; their parents: 4.32 of 6; adolescents: 4.00 of 10; their parents: 3.18 of 5	Weekly email feedback by trained psychologists supervised by an experienced clinical psychologist	NR	Yes
Morgan et al [108], 2017	Web-based CBT-based Cool Little Kids program based on the Cool Little Kids parenting group program addressing anxiety in children	No	8	8 wk	NR	Automated summary emails; telephone support on request by psychologist	12 calls to 11 parents (mean duration 35 min)	Yes
Sanchez et al [109], 2017	Adventures Aboard the S.S. Grin is a web-based game based on the social skills group intervention (DeRosier [128])	No	9 (25 min each)	9 wk	NR	No guidance provided	No guidance provided	No
Calear et al [110], 2016	e-Couch anxiety and worry program: CBT-based program addressing anxiety and worry	No	6 (30-40 min each)	6 wk	2 modules completed: 98%; 4 modules completed: 68%; all 6 modules completed: 45%	No guidance provided (however, classroom teachers assisted with log-ins and supervised participants during time of program completion)	No guidance provided	No
Calear et al [84], 2016	e-Couch anxiety and worry program: CBT-based program mental health condition; e-couch anxiety and worry program: CBT-based program school condition	No; no	6 (30-40 min each); 6 (30-40 min each)	6 wk; 6 wk	NR; NR	No guidance provided (however, participants were supervised to complete the program and assisted if they had questions by teachers and Headspace education officers); no guidance provided (however, classroom teachers assisted with log-ins and supervised participants during time of program completion)	No guidance provided; no guidance provided	No; no
Vigerland et al [94], 2016	CBT-based program addressing anxiety disorders	No	4 for children, 7 for parents	10 wk	Mean number of completed modules: 9.7 (SD 1.8)	Web-based messages and written feedback on worksheets by psychologists and CBT therapists	NR	Yes
Sportel et al [85], 2013	CBM that included interpretation bias and attention bias modification tasks	Yes	20 (40 min each)	10 wk	Mean number of completed sessions: 8.5 (SD 6.7)	No guidance provided (automated emails to complete sessions)	No guidance provided	No
Bowler et al [83], 2012	e-Couch social anxiety program based on CBT principles; CBM with 40 scenarios that relate to persons with social anxiety per module	Yes	4	2 wk	All participants completed all 4 sessions	No guidance provided (however, researcher ensured attendance and compliance with the program in the laboratory)	No guidance provided	No
Tillfors et al [111], 2011	CBT-based self-help manual addressing SAD based on a previously tested program for students	Yes	9	9 wk	Mean number of completed modules: 2.9 (range 1-6)	Feedback on the homework assignment by therapists	NR	No

^a^SAD: social anxiety disorder.

^b^CBT: cognitive behavioral therapy.

^c^NR: not reported.

^d^UP-A: Unified Protocol for Transdiagnostic Treatment of Emotional Disorders in Adolescents.

^e^CBM: cognitive bias modification.

^f^OSCA: Online Social Anxiety Cognitive Therapy for Adolescents.

Most of the interventions (16/22, 73%) were based on CBT principles; some were CBM interventions (3/22, 14%), and some were based on mindfulness (1/22, 5%) or social skills training (1/22, 5%). The interventions either addressed anxiety symptoms or disorders in general or were specifically designed for SAD. SAD-specific interventions [56,86,95,99-101,103,106,111], in particular, incorporated examples of fears and cognitions that are important in SAD (eg, based on the cognitive model [12] or the cognitive behavioral model [14]) or focused on various biases that play a role in SAD [83,85,97].

#### Adherence

Most commonly, adherence was operationalized as the mean number of completed modules per participant or the percentage of participants who completed individual modules [56,83,85,94-97,99,104,105,110,111]. Leigh and Clark [100] reported the average time spent with the intervention, while Nordh et al [103] applied the Internet Intervention Patient Adherence Scale [129]. Only Stapinski et al [104] analyzed the effect of adherence on symptom reduction and found a dose effect with more completed modules resulting in a greater change from baseline to posttreatment assessment.

#### Guidance

The extent of guidance varied greatly across the studies. Of the 22 studies, 9 (41%) were unguided self-help. Among these 9 studies, 6 (67%) provided participants no contact [97-99,105,109,110], while 3 (33%) sent automated reminders or summary emails [85,106,108]. In 2 (9%) of the 22 studies, teachers or researchers were present to ensure that participants engaged with the DMHI, but they gave no feedback on intervention progress [83,84]. Most of the programs individualized their human guidance through asynchronous weekly messages delivered via the program, email, or feedback on submitted worksheets [56,86,94,100,103,104,111]. Some offered scheduled or on-request telephone support during the DMHI period [95,96,100,102-104,108]. Bautista et al [101] offered weekly in-person meetings with a trained undergraduate peer coach. Generally, guidance was provided by trained psychology graduate students under supervision or by trained CBT-therapists or therapists. Some of the studies reported the time spent on guidance; for example, Leigh and Clark [100] recorded an overall mean of 398.67 (SD 59.38) minutes (6.64 hours) of direct communication with participants. Other studies reported average guidance time per week and participant (15-30 minutes) [86,103] or average meeting durations (5-35 minutes) [95,101,108].

### Risk of Bias

**Figure 2:**
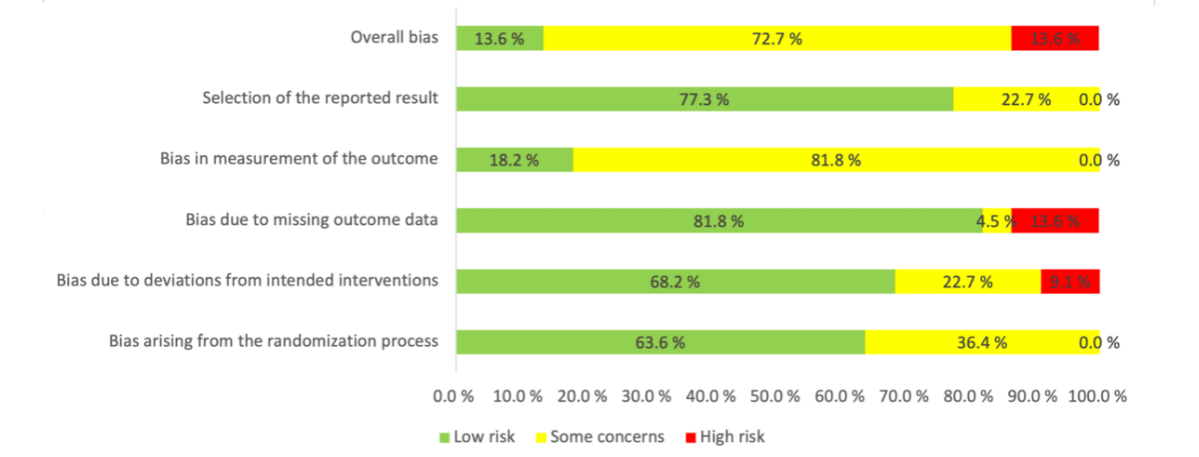
Overall risk-of-bias assessments for all studies.

The risk-of-bias assessment indicated that most of the studies (16/22, 73%) raised some concerns (Figure 2). A main concern was the fact that RCTs investigating psychological interventions did not facilitate adequate masking of participants to the treatment condition. Therefore, only studies that included another active intervention mimicking the initial intervention and in which participants were blinded to the condition [103] were rated as low risk in the *bias in measurement of the outcome* domain. Of the 22 studies, 3 (13.6%) were classified as high risk due to insufficient information on randomization; a lack of analysis estimating the effect of assignment to the intervention group; and high missingness, with absence of measures to address it [86,105,109]. Insufficient information on the randomization process (ie, lack of details on allocation sequence concealment or whether baseline differences between groups were assessed) was reported in 41% (9/22) of the studies. In 32% (7/22) of the studies, there was no information on possible trial deviation or estimation of an effect of assignment to the intervention. Most of the studies (19/22, 86%) followed the intention-to-treat approach and investigated their missing data. Of the 22 studies, 17 (77.3%) reported their plan of analysis, and the reporting of results was transparent and complete. The risk-of-bias assessments for all studies are presented in Multimedia Appendix 3 [56,83-86,94-106,108-111].

### Effect of DMHIs on Social Anxiety Symptoms

**Figure 3:**
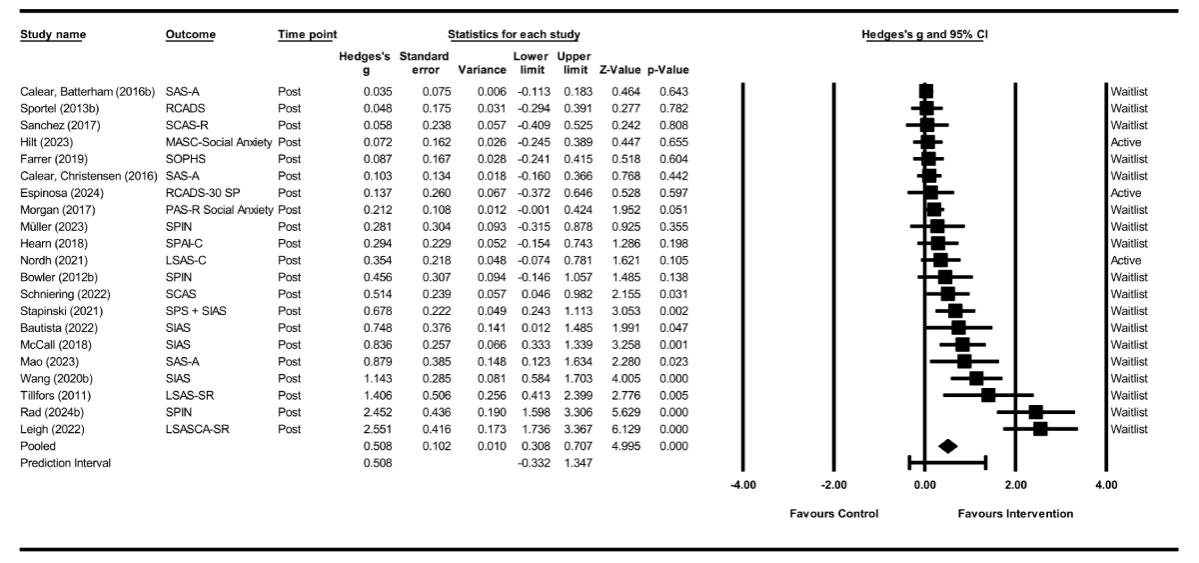
Forest plot of all studies included in the main outcome meta-analysis with the pooled effect size from the random-effects model. LSAS-C: Liebowitz Social Anxiety Scale, child version; LSAS-SR: Liebowitz Social Anxiety Scale–Self-Report; LSASCA-SR: Liebowitz Social Anxiety Scale for Children and Adolescents, self-report version; MASC: Multidimensional Anxiety Scale for Children; PAS-R: Preschool Anxiety Scale, revised; RCADS: Revised Children’s Anxiety and Depression Scale; SAS-A: Social Anxiety Scale for Adolescents; SCAS-R: Spence Children's Anxiety Scale, revised; SIAS: Social Interaction Anxiety Scale; SOPHS: Social Phobia Screener; SPAI-C: Social Phobia and Anxiety Inventory for Children; SPIN: Social Phobia Inventory.

The random-effects model yielded an overall effect size in favor of DMHIs compared to any control condition at the postintervention assessment (Hedges *g*=0.508, 95% CI 0.308-0.707; Figure 3 [83-86,94-111]). Sensitivity analyses were performed (1) excluding studies with the highest [100] and lowest [84] effect sizes, (2) excluding studies with participants aged >25 years [83,86,105,106], and (3) including studies in which all participants had a diagnosis of SAD [86,95,99,100,103,107,111]. Two additional analyses were performed to examine between-group effect sizes at follow-up: one using data collected 3 to 6 months after the postintervention assessment and another using data collected 12 months after the postintervention assessment. A detailed overview is presented in Table 4.

**Table 4:** Effect sizes of digital mental health interventions compared to control groups, based on a random-effects model at the postintervention assessment unless otherwise specified.

						N_co_^a^			Effect size			Test of null (2-tailed)						95% prediction interval			Between-study							Heterogeneity statistics													NNT^b^
									Hedges g (SE; 95% CI)			*Z*			*P* value						Tau			Tau squared			Q (*df*)					*P* value				*I* ^2^					
Overall effect						21			0.508 (0.104; 0.308 to 0.707)			4.995			<.001			−0.332 to 1.347			0.388			0.151			100.164 (20)					<.001				80.033				3.6	
**Sensitivity analyses**																																									
	Highest effect size removed				20			0.414 (0.088; 0.242 to 0.587)			4.705			<.001			−0.262 to 1.091			0.310			0.096			69.414 (19)					<.001				72.628				4.3		
	Lowest effect size removed				20			0.551 (0.111; 0.333 to 0.769)			4.963			<.001			−0.358 to 1.461			0.418			0.175			88.354 (19)					<.001				78.496				3.3		
	Follow-up: 3-6 mo				12			0.378 (0.110; 0.162 to 0.594)			3.427			<.001			−0.366 to 1.122			0.315			0.099			50.871 (11)					<.001				78.376				4.8		
	Follow-up: 12 mo				2			0.064 (0.086; −0.105 to 0.233)			0.748			.46			—^c^			0.000			0.000			0.168 (1)					.68				0.000				27.7		
	Participants aged >25 y omitted				17			0.485 (0.113; 0.264 to 0.706)			4.306			<.001			−0.373 to 1.343			0.387			0.149			83.418 (16)					<.001				80.820				3.7		
	Only participants with a diagnosis of SAD^d^				7			1.149 (0.331; 0.510 to 1.798)			3.473			.001			−1.084 to 3.383			0.803			0.646			46.931 (6)					<.001				87.215				1.7		
**Subgroup analyses**																																									
	**Study characteristics**																																								
		**Control condition**																											10.561 (1)				.001								
			Waitlist	18			0.576 (0.119; 0.343 to 0.809)			4.842			<.001			−0.363 to 1.515			0.427			0.182			98.592 (17)					<.001				82.757				3.2			
			Active	9			0.141 (0.061; 0.021 to 0.261			2.310			.02			—			0.000			0.000			4.068 (8)					.85				0.000				12.6			
		**Risk-of-bias assessment**																											1.554 (2)				.46								
			Low risk	3			0.201 (0.117; −0.029 to 0.431)			1.715			.09			—			0.000			0.000			1.417 (2)					.49				0.000				8.9			
			Some concerns	15			0.622 (0.134; 0.359 to 0.885)			4.633			<.001			−0.371 to 1.614			0.439			0.193			87.152 (14)					<.001				83.936				2.9			
			High risk	3			0.399 (0.312; −0.213 to 1.011)			1.277			.20			−6.972 to 7.770			0.489			0.239			11.344 (2)					.003				82.370				4.5			
		**Age group**																											7.042 (2)				.03								
			Children (aged <10 y)	2			0.185 (0.099; −0.008 to 0.379)			1.877			.06			—			0.000			0.000			0.346 (1)					.56				0.000				9.6			
			Adolescents (aged 11-17 y)	12			0.575 (0.156; 0.270 to 0.880)			3.697			<.001			−0.521 to 1.672			0.467			0.218			76.542 (11)					<.001				85.629				3.2			
			Young adults (aged >18 y)	7			0.578 (0.154; 0.276 to 0.879)			3.754			<.001			−0.301 to 1.457			0.305			0.093			14.484 (6)					.03				58.576				3.2			
	**Intervention characteristics**																																								
		**Psychological principle**																											6.302 (1)				.01								
			CBT^e^	16			0.610 (0.127; 0.361 to 0.859)			4.800			<.001			−0.361 to 1.580			0.434			0.189			93.551 (15)					<.001				83.966				3.0			
			Other	5			0.176 (0.117; −0.052 to 0.405)			1.512			0.729			−0.376 to 0.729			0.129			0.017			5.285 (4)					.26				24.307				10.1			
		**SAD-specific intervention**																											11.034 (1)				.001								
			Yes	12			0.878 (0.206; 0.469 to 1.278)			4.231			<.001			−0.615 to 2.362			0.635			0.403			63.447 (11)					<.001				82.663				2.2			
			No (transdiagnostic intervention)	9			0.195 (0.061; 0.040 to 0.277)			2.622			.009			−0.104 to 0.422			0.093			0.093			11.150 (8)					.19				28.250				9.1			
		**Guidance**																											6.065 (1)				.01								
			No	11			0.271 (0.096; 0.083 to 0.458)			2.834			.005			−0.301 to 0.842			0.234			0.055			27.308 (10)					.002				63.381				6.6			
			Yes	10			0.825 (0.204; 0.425 to 1.224)			4.049			<.001			−0.567 to 2.216			0.568			0.323			58.760 (9)					<.001				84.683				2.3			
		**Parental involvement**																											0.670 (1)				.41								
			Yes	4			0.281 (0.084; 0.117 to 0.445)			3.355			.001			—			0.000			0.000			1.476 (3)					.69				0.000				6.4			
			No	10			0.602 (0.184; 0.242 to 0.962)			3.275			.001			−0.644 to 1.847			0.508			0.258			74.031 (9)					<.001				87.843				3.0			

^a^N_co_: number of comparisons (ie, the number of primary studies included in the meta-analytical evaluation and subgroup analyses).

^b^NNT: number needed to treat.

^c^Not available.

^d^SAD: social anxiety disorder.

^e^CBT: cognitive behavioral therapy.

### Subgroup Analyses

In subgroup analyses, we investigated the moderating effect of the control condition, risk of bias, age group, underlying psychological principles, the focus of the intervention on SAD or transdiagnostic anxiety symptoms, guidance, and parental involvement. Except for the subgroup analysis comparing the effect of control conditions, both active and inactive conditions were included in all analyses. The effect size was significantly greater when the DMHI was compared to a waitlist (Hedges g=0.576, 95% CI 0.343-0.809) than to another active intervention (Hedges g=0.101, 95% CI 0.021-0.261). Furthermore, DMHIs based on CBT (Hedges g=0.610, 95% CI 0.361-0.859) were significantly more effective than DMHIs based on other therapeutic interventions (Hedges g=0.176, 95% CI −0.052 to 0.405; ie, CBM). DMHIs including therapeutic elements specifically addressing SAD (Hedges g=0.878, 95% CI 0.469-1.278) were significantly more effective than DMHIs addressing transdiagnostic anxiety symptoms (Hedges g=0.195, 95% CI 0.040-0.277). Guided self-help (Hedges g=0.825, 95% CI 0.425-1.224) was more efficacious than unguided self-help (Hedges g=0.271, 95% CI 0.083-0.458). Effect sizes differed significantly across age groups (children: Hedges g=0.185, 95% CI −0.008 to 0.379; adolescents: Hedges g=0.575, 95% CI 0.270-0.880; and young adults: Hedges g=0.578, 95% CI 0.276-0.879), with the smallest and nonsignificant effect observed in children. No significant difference was found for DMHIs with (Hedges g=0.281, 95% CI 0.117-0.445) or without (Hedges g=0.602, 95% CI 0.242-0.962) parental involvement. The computed effect sizes for different levels of risk of bias were not significantly different from each other (low risk: Hedges g=0.201, 95% CI −0.029 to 0.431; some concerns: Hedges g=0.622, 95% CI 0.359-0.885; and high risk: Hedges g=0.399, 95% CI −0.213 to 1.011); however, while the 95% prediction interval for the low-risk and some-concerns subgroup analyses were small, the one in the high-risk bias subgroup analysis spanned from –6.972 to 7.770. A complete overview is presented in Table 4, and all forest plots are included in Multimedia Appendix 4 [83-86,95-111].

### Meta-Regression of Age and Symptom Severity

Age and baseline symptom severity of social anxiety were introduced as moderators in the main analysis. The meta-regression indicated that age had no significant effect on the efficacy of DMHIs on social anxiety (𝛽=.038, 95% CI –0.003 to 0.079; *P*=.07). Higher baseline symptom severity increased the effect size significantly (β=.014, 95% CI 0.005-0.023; *P*=.002).

### Publication Bias

**Figure 4:**
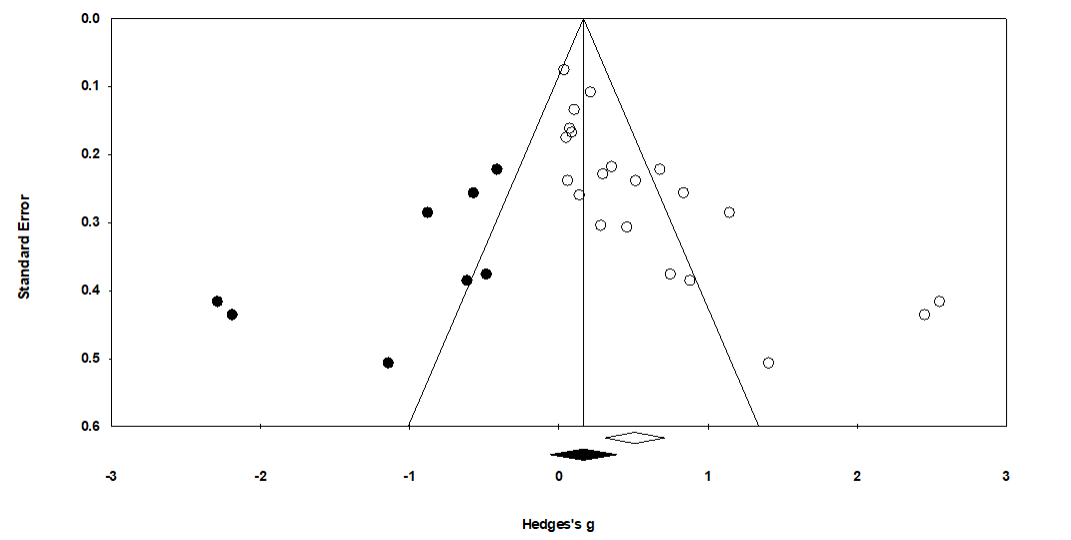
Funnel plot of all studies included in the meta-analysis, showing the corrected mean effect (black diamond) and imputed effect sizes (black dots).

A visual inspection of the funnel plot suggested potential publication bias: the SEs were not symmetrically distributed around the overall mean (Figure 4). Both the Begg and Mazumdar rank correlation test and the Egger linear regression test of the intercept were significant (*P*<.001 for both), indicating an underlying publication bias. When adjusting for missing studies using the trim-and-fill approach proposed by Duval and Tweedie [90] in the random-effects model, the resulting effect size was Hedges *g*=0.506 (95% CI 0.308-0.707).

## Discussion

### Principal Findings

The meta-analysis evaluated the efficacy of DMHIs on SAD in young people. Across 21 RCTs and 4196 participants, we found a small to medium pooled effect size in favor of DMHIs compared to both inactive and active control conditions assessed after the intervention. The effect size remained after taking the publication bias into account. Notably, the treatment gains diminished by the 3- to 6-month follow-up and were no longer significant at the 12-month follow-up. Subgroup analyses revealed that DMHIs based on CBT, including SAD-specific elements, and supported by human guidance implemented in adolescents and young adults resulted in higher pooled effect sizes compared to other psychological interventions, transdiagnostic interventions for several anxiety disorders, and unguided self-help programs in children. Mean age and parental involvement had no significant effect on the outcome.

### Overall Effects in Comparison to Other Meta-Analyses

Compared to other meta-analyses of DMHIs addressing anxiety disorders, the overall effect size found in this meta-analysis was smaller. In a similarly aged sample but one with mixed anxiety disorders, a meta-analysis found a large pooled effect size (Hedges *g*=0.68) [45]. Even in children and adolescents at risk for an anxiety disorder (ie, subthreshold of diagnosis), where smaller effect sizes would be expected, a large pooled effect size (SMD 0.77) was found [44]. Possible reasons for the smaller effect compared to previous meta-analyses could be the large heterogeneity between the included studies regarding the interventions, the samples, and the study designs. Another reason could be the fact that despite the focus of this meta-analysis on samples with SAD, the primary studies included a broad range of mental health problems or disorders (eg, other anxiety disorders and depression). Consequently, study participants did not necessarily have social anxiety. In other meta-analyses, either a measure assessing various anxiety disorders was defined as the primary outcome, or the interventions and samples were primarily geared toward SAD. The sensitivity analysis in this meta-analysis showed that when only studies with participants with a SAD diagnosis were included, the calculated effect size was large (Hedges *g*=1.15). Meta-analytic evidence from face-to-face settings for children and adolescents diagnosed with SAD only also yielded a larger effect (Hedges *g*=0.71), which increased at follow-up [22]. Similar effect sizes [130] were found in iCBT for adults with SAD (Hedges *g*=0.55 [130]; Hedges *g*=0.76 [131]). In line with these findings, the moderator analyses showed that there are, in some cases, large differences between the effect sizes of the subgroups. These differences are discussed in the following subsections.

### Psychological Principles of Effective DMHIs

The superiority of CBT-based treatments over other treatments, such as internet-delivered CBM or mindfulness exercises, is also reflected in other studies [58,132]. However, most treatments for young people with SAD are based on CBT, and the evidence for other treatments is still limited [133]. Interestingly, SAD-specific treatments, including CBT and CBM, outperformed treatments addressing anxiety in general. This finding contradicts results from RCTs directly comparing 2 treatments (transdiagnostic anxiety treatment vs a SAD-specific approach [55,56]) but aligns with other evidence suggesting that adolescents with SAD have poorer outcomes than those with other anxiety disorders [53,54,134]. This finding is quite striking because DMHIs addressing SAD in adults have an extensive evidence base, with RCTs demonstrating efficacious interventions based on CBT, psychodynamic therapy, and acceptance-based interventions [39] and meta-analytic evidence supporting iCBT to be as efficacious as face-to-face CBT [42]. In young people, the question remains as to which treatment components have a generic effect in anxiety disorders and which would improve the treatment effect in SAD and thus indicate a disorder-specific treatment. Treatment components specific to SAD are theoretically discussed in etiological and maintenance models of SAD [12-14]. Empirical evidence remains limited regarding whether these components are specifically important for young people with SAD [21]. It would be valuable to test both transdiagnostic anxiety and SAD-specific components individually in interventions. Compared to traditional RCTs, study designs such as factorial or leapfrog designs would be better suited to evaluate the efficacy of individual components and determine the most effective combination for intervention development [135-138].

### Supporting Factors in DMHI Implementation for Young People

Similar to previous evidence, moderator analyses supported the efficacy of guided DMHIs but not unguided DMHIs [59]. Notably, there was great variety in the extent of human guidance—from summaries of exercises sent to participants via chat or email to regular telephone contact. The amount of support was quantified only in a minority of the trials (6/22, 27%). Furthermore, while some guidance formats reflected strategies to improve engagement with the intervention, the guidance of other trials could be viewed as additional treatment, aligning more with blended therapy. To investigate how to optimize the amount, the content, or the delivery setting of guidance for young people, future studies should be more detailed in their report of the guidance format and its intended purpose and may even compare different support strategies for young people [139]. Possible results could streamline future implementation research and support the use of, and continuous engagement with, digital approaches.

Another aspect that varied considerably across the studies was parental involvement. In the study by Morgan et al [108], the intervention was only directed at parents, while other studies included specific parent information or modules that complemented the children’s modules [56,94,102,103] or only addressed the young people. Research to date shows an inconclusive picture of how parents can be best involved in treatments to increase their overall effectiveness compared to interventions meant only for children or adolescents [140]. Comparable to our results, other meta-analyses also found no additional effect of parental involvement [141-143]. Especially in adolescence and young adulthood, the social context relevant to anxiety-inducing situations lies primarily outside the parental home (eg, at school and with peers). Therefore, the involvement of parents might be less relevant for symptom reduction. Nevertheless, for young people, parents play an important role in accessing mental health services [144,145] and could therefore be a crucial supporting factor in their children’s adherence to the intervention [146].

Adherence was defined and reported, if at all, in great variety in the included studies. Most commonly, completed modules and time spent in the DMHI were reported; however, their effect on treatment outcome was rarely examined. Although these metrics offer some insight into DMHI use, they do not provide any information about reasons for nonadherence. Previous efforts have sought to explain nonuse of DMHIs or treatment. In adult samples, a proposed explanation is the “good enough” effect—participants may discontinue a DMHI after achieving their personal goals [147]. A qualitative examination of adolescents who stopped treatment for depression identified a similar group, along with 2 additional types: the *dissatisfied*, who did not find the intervention helpful; and the *troubled*, who reported a lack of stability in their lives that hindered engagement with an intervention [148]. Future research should report a minimum of adherence parameters (eg, completed modules and time spent in the program) and might even add reasons for nonadherence. Furthermore, it would be important to report the analysis of whether adherence influenced the outcome within the publication reporting the main results of the RCT. With this information, studies could be better compared, and the influence of adherence on the outcome could be assessed meta-analytically [149].

### Moderators of Treatment Efficacy

Regarding potential moderators, age did not emerge as a moderator of treatment efficacy in the meta-regression analysis, but the subgroup analysis reported significantly higher effect sizes for adolescents and young adults compared to children. Likewise, other meta-analyses have indicated that older adolescents profit more from DMHIs than children [37]. However, the small number of studies including child samples in this meta-analysis could also be a reason for this result. Furthermore, although the influence of age was taken into account in this meta-analysis—through meta-regression (mean age), subgroup analyses (age groups), and sensitivity analyses excluding studies with participants aged >25 years—many of the primary studies included wide age ranges and did not report outcomes separately by age group. This highlights the need to replicate these findings in future studies.

Baseline symptom severity moderated treatment outcome with a small effect. In other studies, larger treatment effects were found for adults with higher initial symptom severity [150]. The evidence in childhood and adolescence is mixed: some meta-analyses or studies found a significant effect, while others did not [151]. Therefore, this result is in line with the overall mixed picture.

### Designing and Implementing DMHIs in the Future

This review included DMHIs that were developed primarily in high-income countries and provided in English, Swedish, Chinese, Persian, Spanish, or Dutch. Furthermore, only a few undertook efforts to match the socioeconomic composition of the study with that of the home country [56,100]. A recurring argument in favor of DMHIs is that they offer help at low cost with a low threshold and high accessibility to young people from nonurban and lower socioeconomic groups [38,152,153]. However, this presumption can only be realized if measures are taken to design DMHIs for diverse target groups; evaluate them in different demographic groups; and support their implementation, particularly in those groups that otherwise have little access to mental health care. To achieve this, young people need to be actively involved in designing interventions and validation studies, recruiting participants for trials, and communicating about and implementing digital self-help options [133,154].

### Limitations and Implications for Future Research

This review and meta-analysis have some limitations, and the findings should be considered thoughtfully. First, the meta-analysis and included subgroup analyses were based on a small number of studies, and the results should be interpreted with caution. The limited number of studies precluded an examination of the efficacy of specific intervention components (eg, psychoeducation, exposure therapy, social skills training, CBM, or relaxation and mindfulness exercises) or the differentiation of effects between diagnostic categories (subclinical vs clinical). Most of the studies included participants with elevated scores in social anxiety screenings based on self-reports, and only a few of the identified prevention studies made diagnostic efforts to distinguish youths with subclinical social anxiety symptoms from those with a previous or current SAD diagnosis. Thus, prevention and treatment studies could not be categorized reliably to investigate potential differential treatment effects in subgroup analyses. Moreover, only 2 studies provided long-term follow-up data, resulting in limited information on the sustainability of treatment effects. Next, the meta-analysis encompassed a broad age range, spanning distinct developmental stages and diverse target groups; for instance, while adolescents are typically the primary end users of digital interventions, parents or legal guardians generally serve this role for preschool-aged children, who often lack the cognitive and emotional capacities to engage with such interventions independently. To explore potential age-related effects, subgroup analyses were conducted across 3 age categories (ie, children, adolescents, and young adults). However, these analyses were limited by the small number of primary studies within each subgroup and did not account for developmental differences within or between age groups. Given the substantial developmental variability that exists within these broad categories, future research should examine potential moderator effects using more granular age distinctions and developmental profiles, particularly in terms of cognitive, emotional, and social competencies.

Furthermore, factors influencing efficacy, such as adherence, were inadequately reported in primary studies, preventing their incorporation into the meta-analysis, for instance, in a meta-regression. Finally, the methodological quality of the studies varied in terms of transparency in randomization, masking procedures, and the consideration of appropriate statistical procedures.

Therefore, future intervention studies should be methodologically more rigorous; include thorough diagnostic procedures; provide long-term follow-up data; and report on potential moderators such as adherence, parental involvement, guidance, and participant characteristics in more detail. Moreover, more details on how access to the modules was managed would be interesting because it may have an impact on participant adherence as well as intervention efficacy. Furthermore, future studies should aim to target a socioeconomically diverse study population and report on demographic information that may influence the availability and efficacy of treatments (eg, gender, age, country, urbanicity, socioeconomic status, culture, and minoritized status [155]). This additional information could inform future meta-analyses or, preferably, meta-analyses of individual participant data that do not rely on summary statistics to investigate subgroup effects.

### Conclusions

In conclusion, while further research is necessary to provide conclusive evidence on the efficacy of DMHIs for young people, our systematic review and meta-analysis show their potential to alleviate SAD symptoms. The preliminary findings underscore the importance of future research efforts aimed at clarifying the specific treatment components that render DMHIs effective, optimizing human guidance strategies and individualizing interventions to enhance therapeutic outcomes. Moreover, our study highlights the need for greater inclusivity in DMHIs and research design, emphasizing the need for future studies to encompass participants from all socioeconomic backgrounds. By addressing these considerations, DMHIs hold promise in bridging the care gap and democratizing access to psychotherapeutic interventions for all young people, thus fostering mental health equity in the future.

## Appendix

Multimedia Appendix 1 PRISMA (Preferred Reporting Items for Systematic Reviews and Meta-Analyses) checklist.

Multimedia Appendix 2 Search strings used for the systematic literature review.

Multimedia Appendix 3 Risk-of-bias assessments for all studies across all 5 domains.

Multimedia Appendix 4 Forest plots for all meta-analyses.

## Abbreviations

CBM: cognitive bias modification
CBT: cognitive behavioral therapy
DMHI: digital mental health intervention
iCBT: internet-delivered cognitive behavioral therapy
PRISMA: Preferred Reporting Items for Systematic Reviews and Meta-Analyses
RCT: randomized controlled trial
RoB 2: revised Cochrane Risk of Bias Tool for Randomized Trials
SAD: social anxiety disorder
SMD: standardized mean difference
