# Supplementary material for: Digital Mental Health Interventions for the Prevention and Treatment of Social Anxiety Disorder in Children, Adolescents, and Young Adults: Systematic Review and Meta-Analysis of Randomized Controlled Trials
Source: J Med Internet Res. 2025 Jun 12;27:e67067. doi: 10.2196/67067 (PMC12203032; doi:10.2196/67067)
Supplement: Multimedia Appendix 2 [file jmir_v27i1e67067_app2.docx]

Multimedia Appendix 1

Search Strings used for the systematic literature review. Searches were conducted on July 10^th^ 2024.

Ovid

(((("social phobia" or "social anxiety disorder" or "social anxiety" or "socially anxious" or anxiety) and (child* or youth or young or adolescen* or teen* or "emerging adults" or "high-school students") and (internet* or online* or computer* or web* or digital or smartphone or phone* or mobile* or handy or chat or game or app or virtual reality)) adj5 (therapy or therapies or treatment* or intervention* or program* or prevention or CBT)) and ("randomized controlled trial" or "randomised controlled trial" or random*)).mp. [mp=ti, ab, hw, tn, ot, dm, mf, dv, kf, fx, dq, bt, nm, ox, px, rx, an, ui, sy, tc, id, tm, fw, cw, kx, iz]

limit to randomized controlled trial

limit to journal article

remove duplicates

Web of Science

((((TS=("social phobia" OR "social anxiety disorder" OR "social anxiety" OR "socially anxious" OR anxiety)) AND TS=(child* or youth or young or adolescen* or teen* or "emerging adults" or "high-school students"))) AND TS=("internet-delivered therapy" OR "web-based therapy" "internet-based CBT" OR "web-based intervention" OR "internet-based cognitive behavioral therapy" OR "internet-based cognitive behavioural therapy" OR "internet-based cognitive therapy" OR "internet-based behavioral therapy" OR "internet-based behavioural therapy" OR "web-based intervention" "app-based intervention" OR "web-based program*")) AND TS=("randomized controlled trial" or "randomised controlled trial" or random*)

Pubmed

(((("social phobia"[Title/Abstract] OR "social anxiety disorder"[Title/Abstract] OR "social anxiety"[Title/Abstract] OR "socially anxious"[Title/Abstract] OR anxiety[Title/Abstract]) AND (child*[Title/Abstract] OR youth[Title/Abstract] OR young[Title/Abstract] OR adolescen*[Title/Abstract] OR teen*[Title/Abstract] OR "emerging adults"[Title/Abstract] OR "high-school students"[Title/Abstract])) ) AND (internet*[Title/Abstract] OR online*[Title/Abstract] OR computer*[Title/Abstract] OR web*[Title/Abstract] OR digital[Title/Abstract] OR smartphone[Title/Abstract] OR phone*[Title/Abstract] OR mobile*[Title/Abstract] OR handy[Title/Abstract] OR chat[Title/Abstract] OR game[Title/Abstract] OR app[Title/Abstract] OR virtual reality[Title/Abstract])) AND (therapy[Title/Abstract] OR therapies[Title/Abstract] OR treatment*[Title/Abstract] OR intervention*[Title/Abstract] OR program*[Title/Abstract] OR prevention[Title/Abstract] OR CBT[Title/Abstract])

Filters: Randomized Controlled Trial
