# Supplementary material for: Digital Mental Health Interventions for the Prevention and Treatment of Social Anxiety Disorder in Children, Adolescents, and Young Adults: Systematic Review and Meta-Analysis of Randomized Controlled Trials
Source: J Med Internet Res. 2025 Jun 12;27:e67067. doi: 10.2196/67067 (PMC12203032; doi:10.2196/67067)
Supplement: Multimedia Appendix 3 [file jmir_v27i1e67067_app3.docx]

Multimedia Appendix 2

Risk of Bias assessment of all studies over all five domains.
