# Supplementary material for: Digital Mental Health Interventions for the Prevention and Treatment of Social Anxiety Disorder in Children, Adolescents, and Young Adults: Systematic Review and Meta-Analysis of Randomized Controlled Trials
Source: J Med Internet Res. 2025 Jun 12;27:e67067. doi: 10.2196/67067 (PMC12203032; doi:10.2196/67067)
Supplement: Multimedia Appendix 4 [file jmir_v27i1e67067_app4.pptx]

## Slide 1
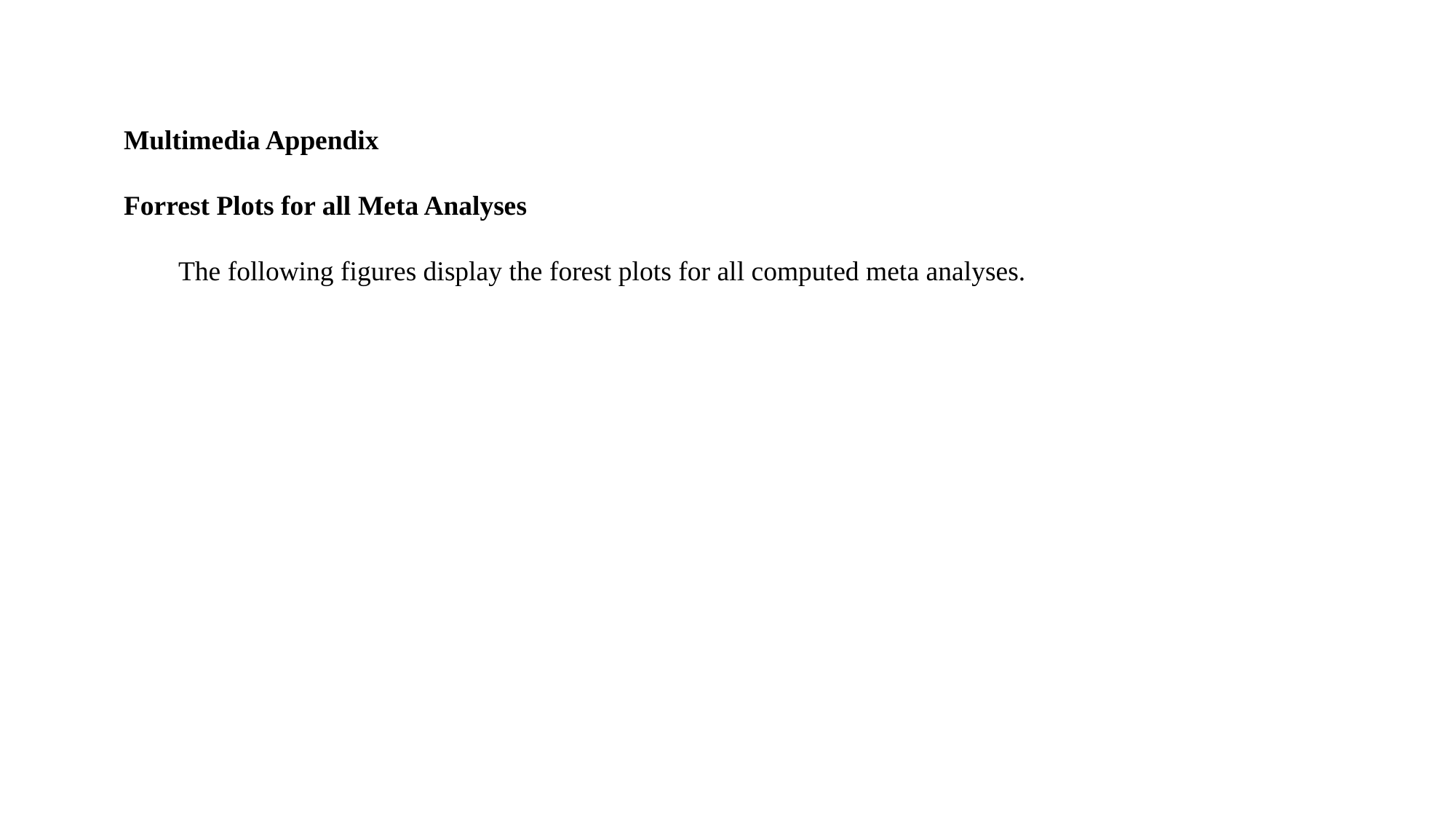

Multimedia Appendix
Forrest Plots for all Meta Analyses
The following figures display the forest plots for all computed meta analyses.

## Slide 2
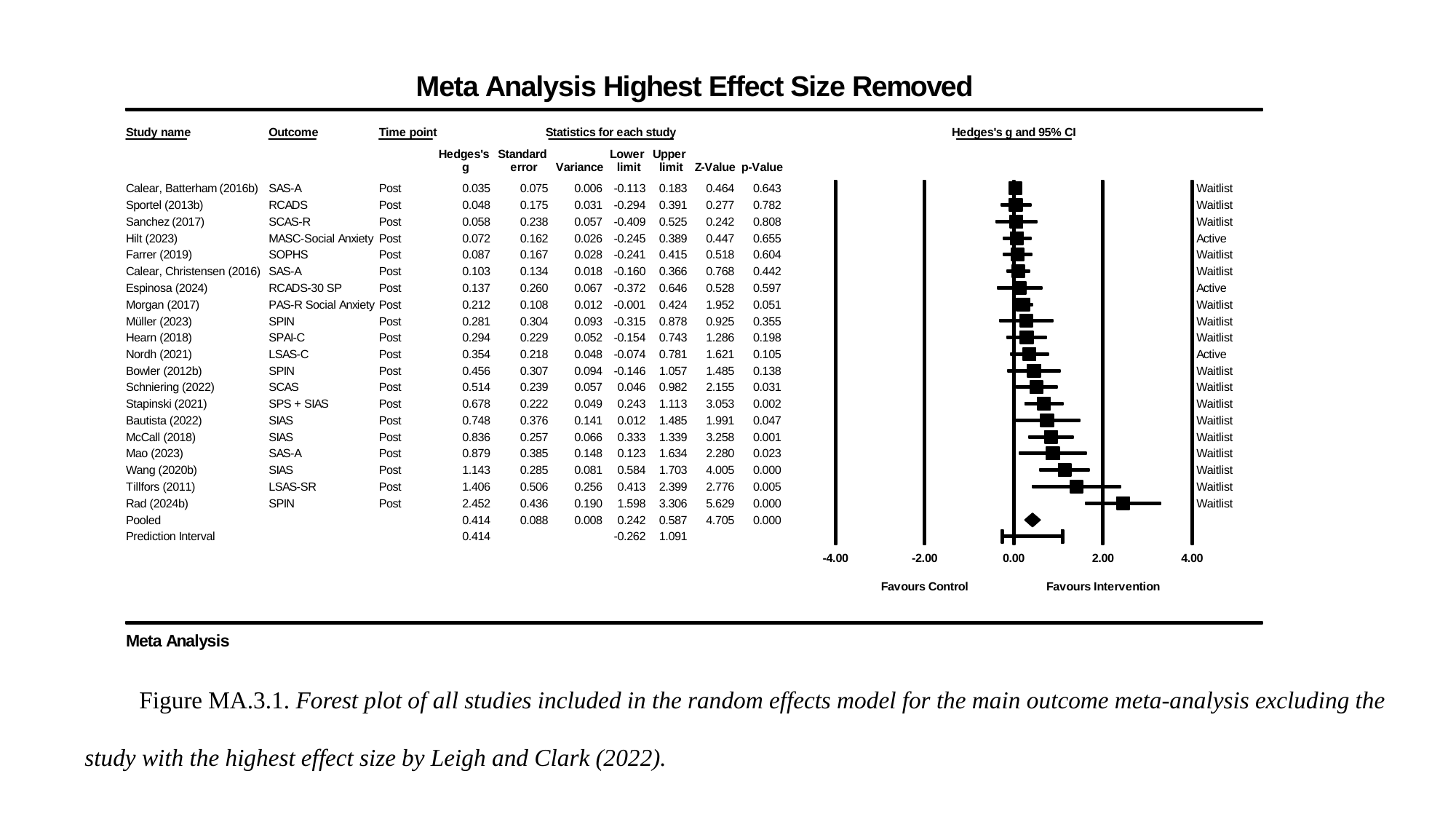

Figure MA.3.1. Forest plot of all studies included in the random effects model for the main outcome meta-analysis excluding the study with the highest effect size by Leigh and Clark (2022).

## Slide 3
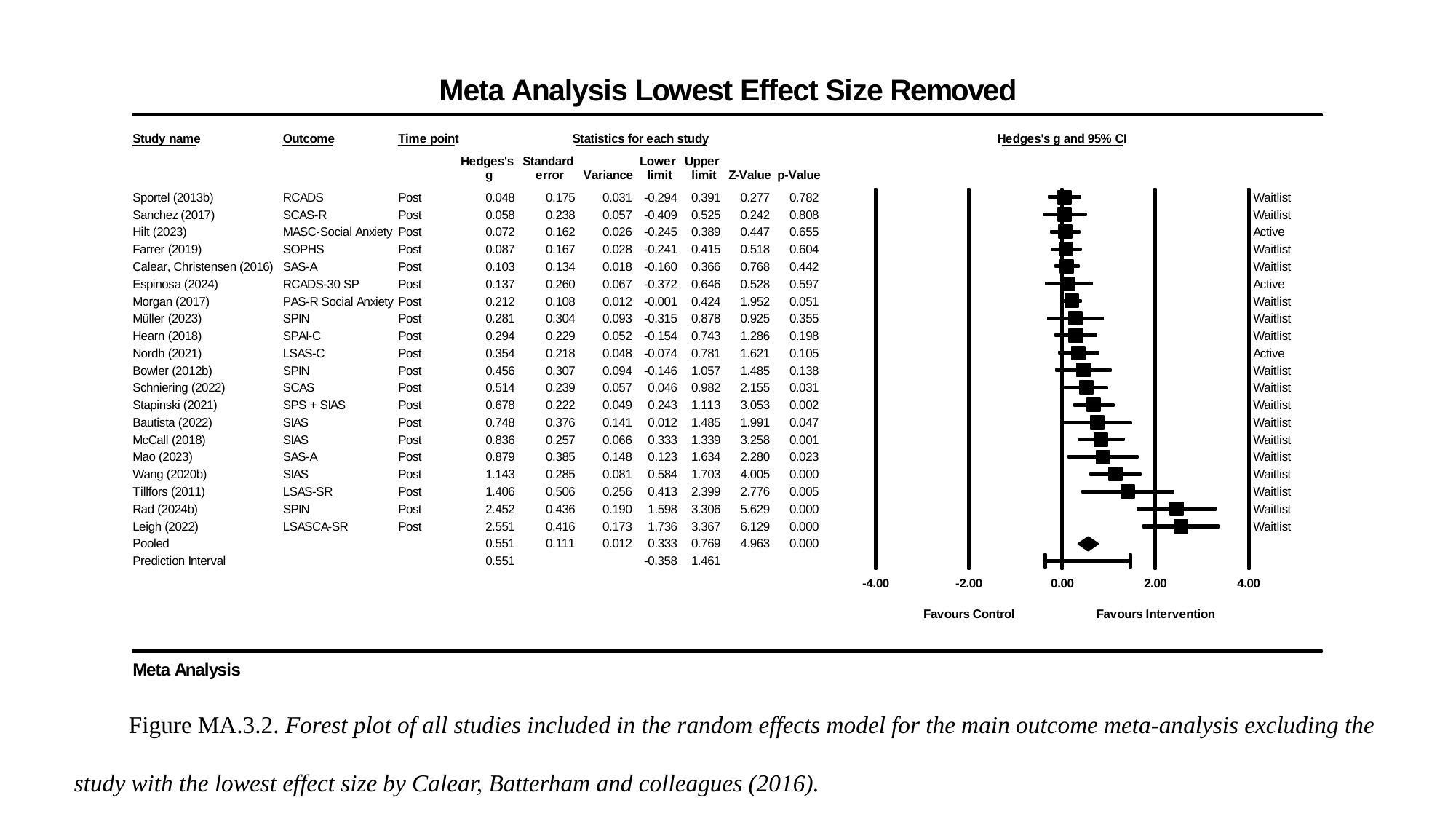

Figure MA.3.2. Forest plot of all studies included in the random effects model for the main outcome meta-analysis excluding the study with the lowest effect size by Calear, Batterham and colleagues (2016).

## Slide 4
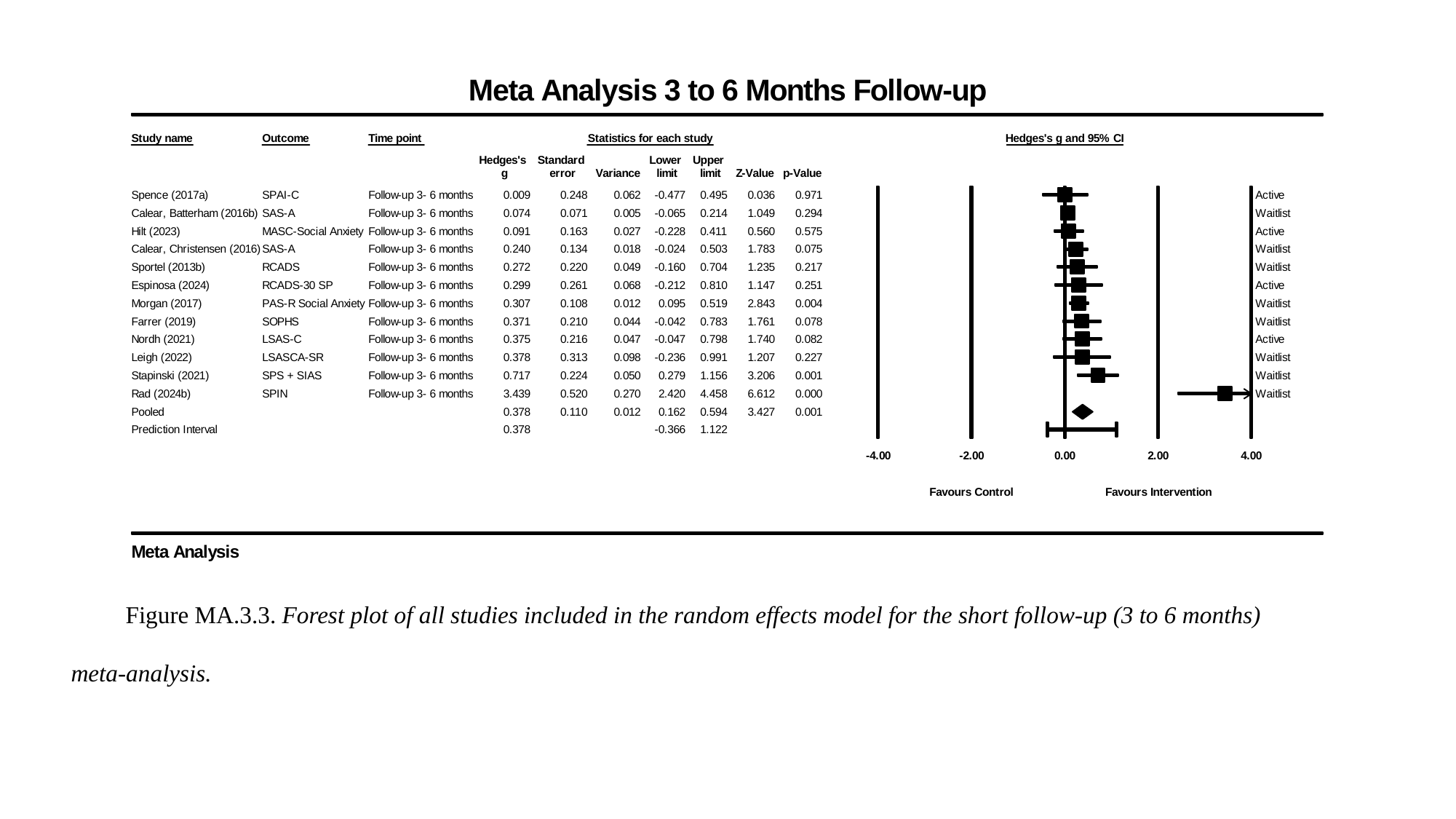

Figure MA.3.3. Forest plot of all studies included in the random effects model for the short follow-up (3 to 6 months) meta-analysis.

## Slide 5
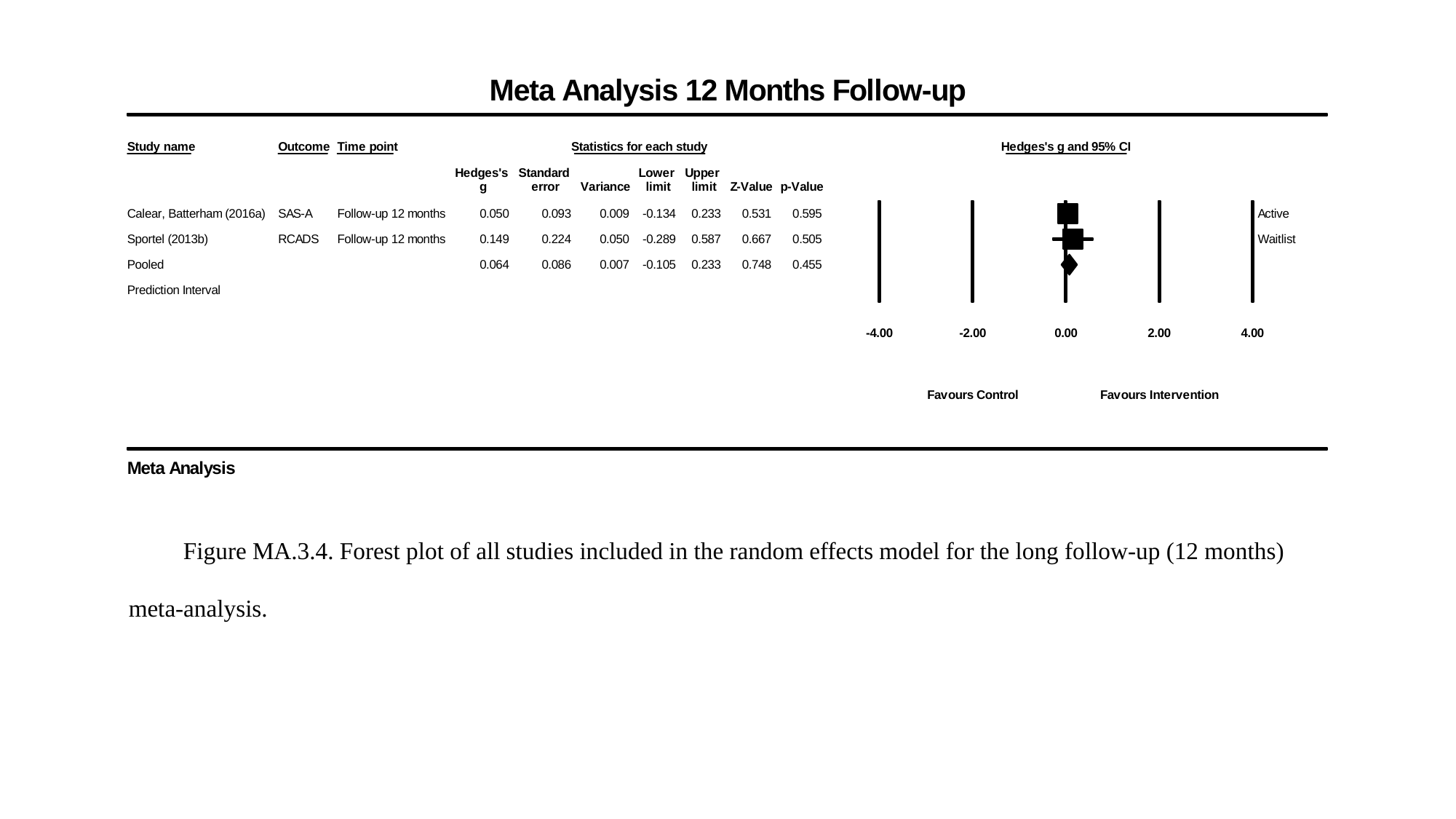

Figure MA.3.4. Forest plot of all studies included in the random effects model for the long follow-up (12 months) meta-analysis.

## Slide 6
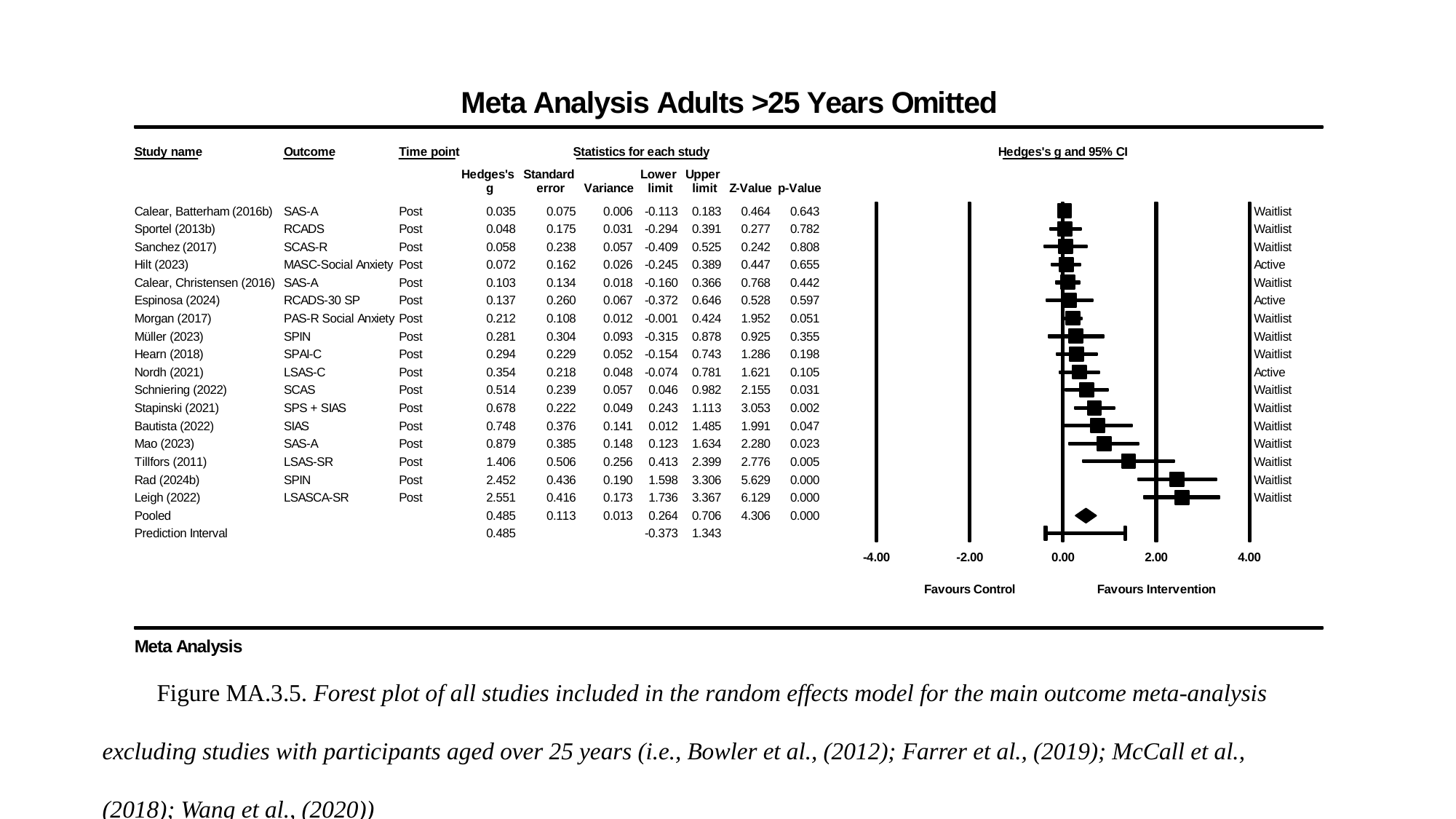

Figure MA.3.5. Forest plot of all studies included in the random effects model for the main outcome meta-analysis excluding studies with participants aged over 25 years (i.e., Bowler et al., (2012); Farrer et al., (2019); McCall et al., (2018); Wang et al., (2020))

## Slide 7
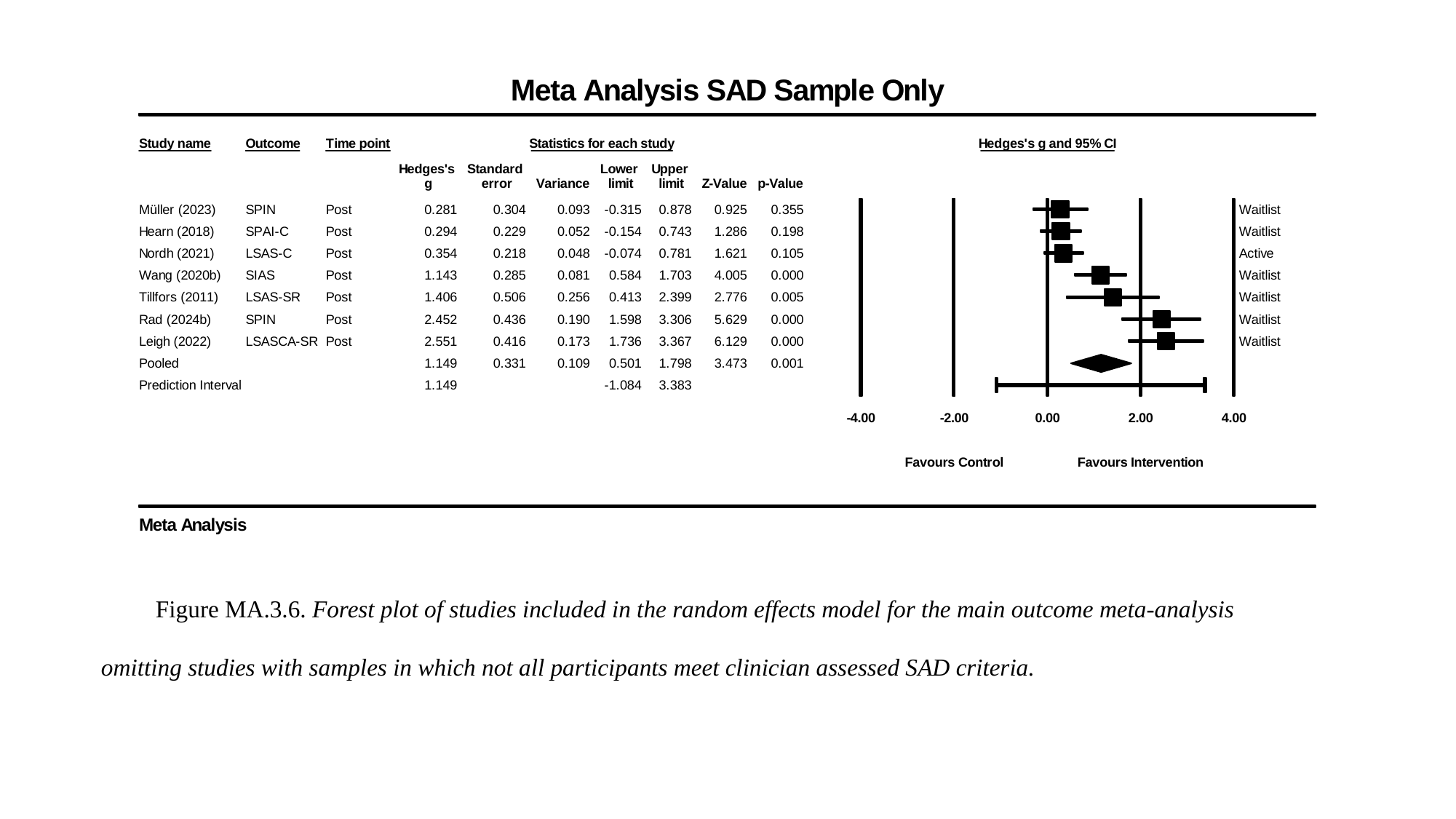

Figure MA.3.6. Forest plot of studies included in the random effects model for the main outcome meta-analysis omitting studies with samples in which not all participants meet clinician assessed SAD criteria.

## Slide 8
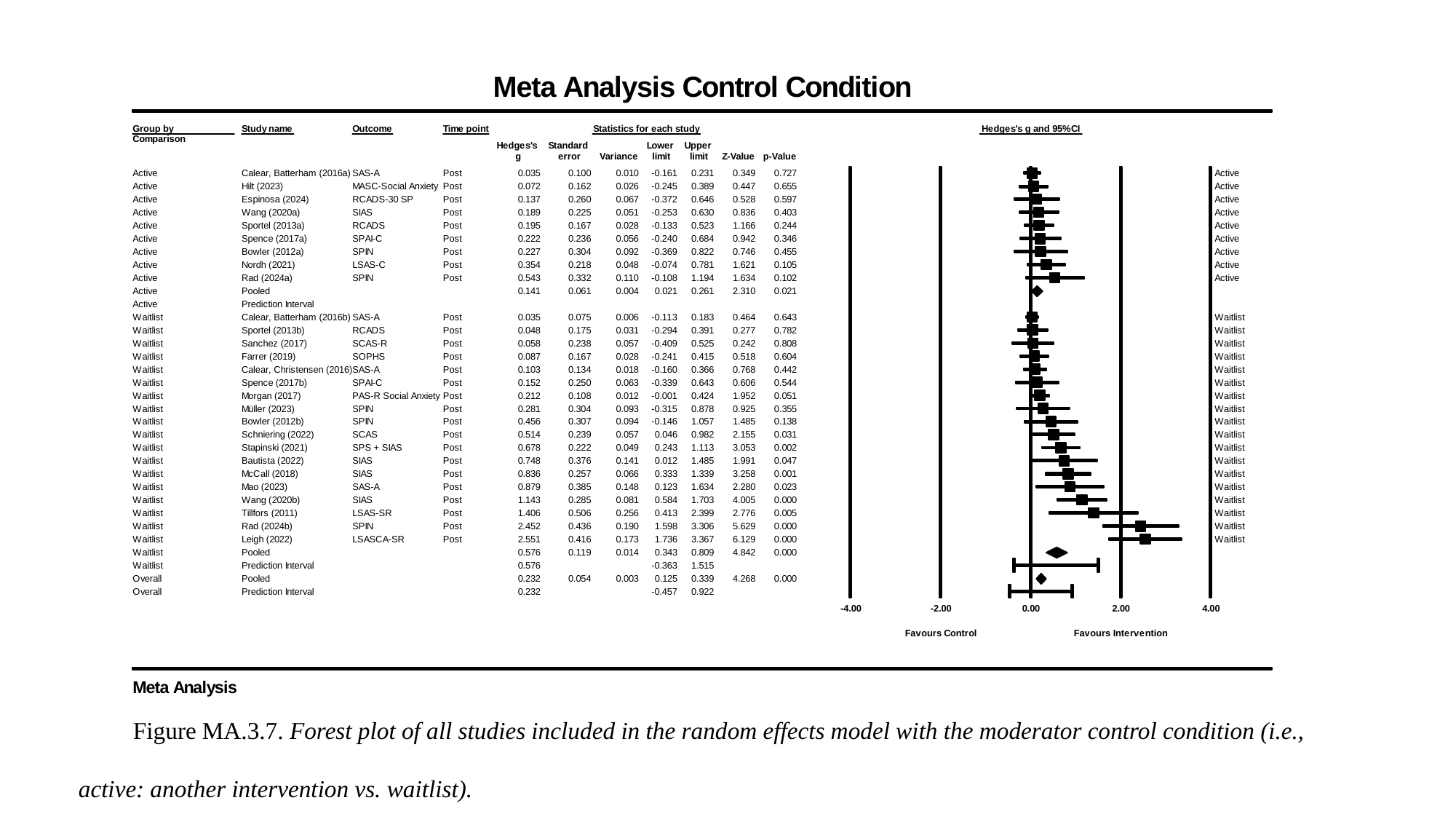

Figure MA.3.7. Forest plot of all studies included in the random effects model with the moderator control condition (i.e., active: another intervention vs. waitlist).

## Slide 9
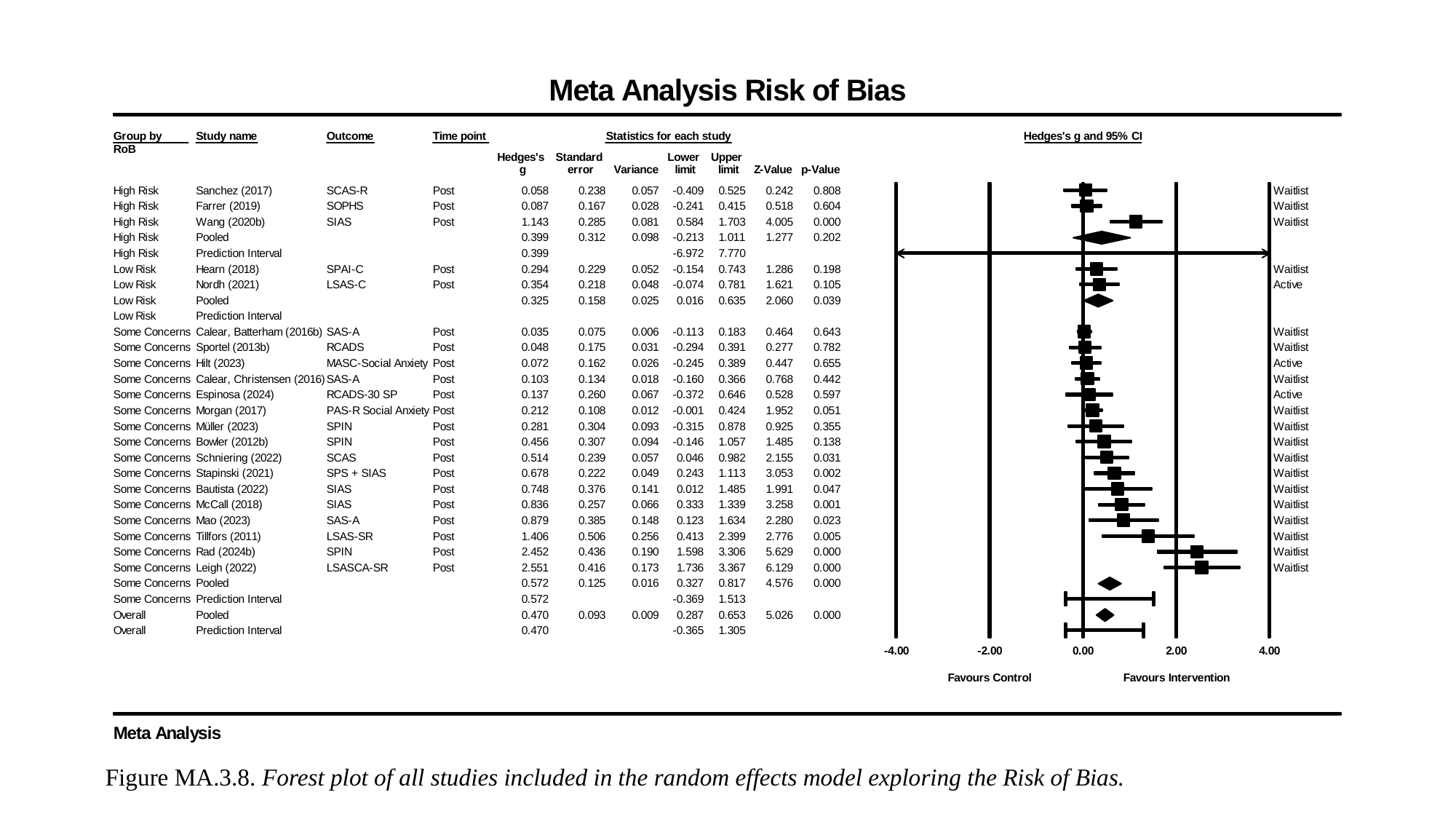

Figure MA.3.8. Forest plot of all studies included in the random effects model exploring the Risk of Bias.

## Slide 10
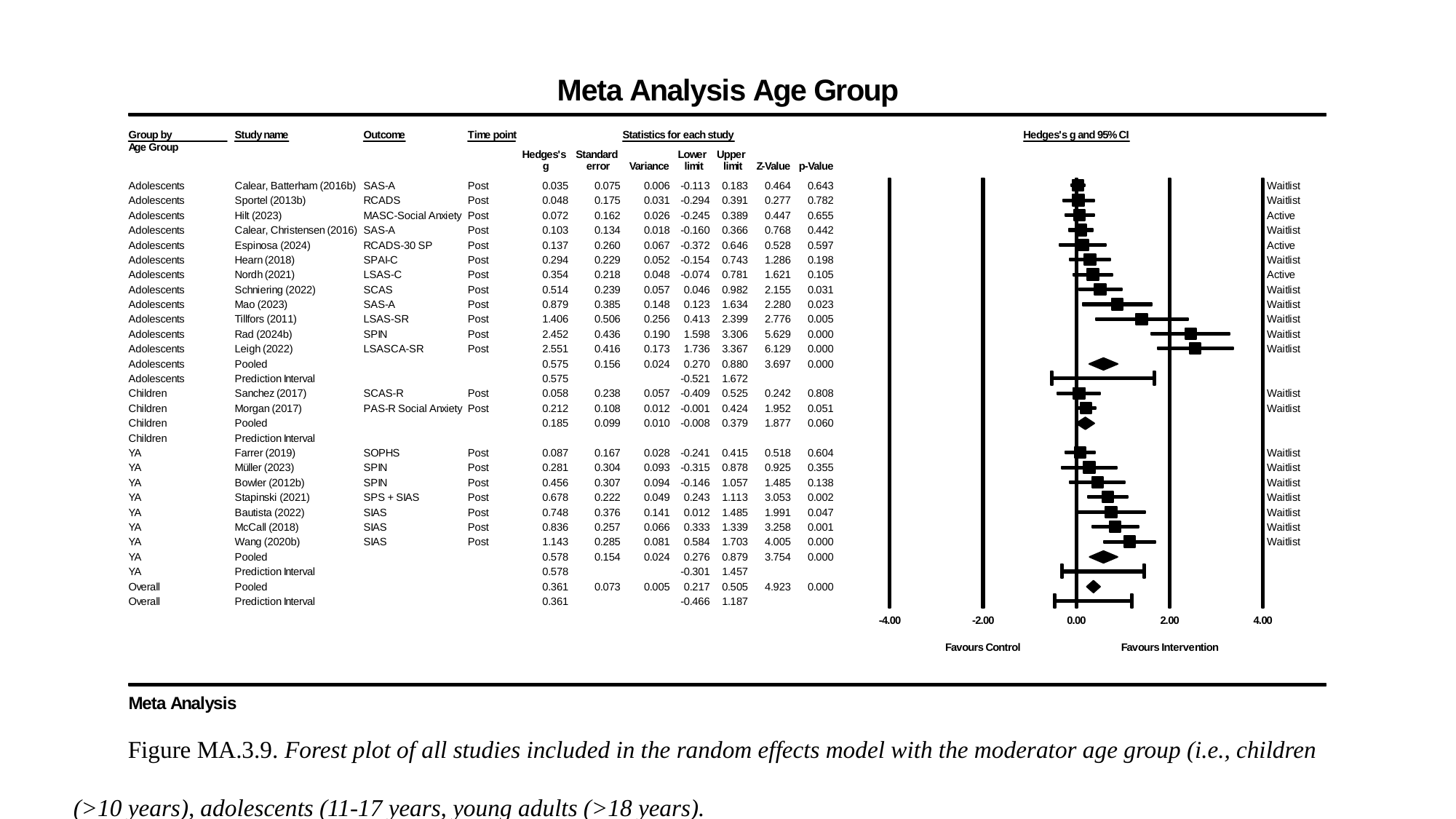

Figure MA.3.9. Forest plot of all studies included in the random effects model with the moderator age group (i.e., children (>10 years), adolescents (11-17 years, young adults (>18 years).

## Slide 11
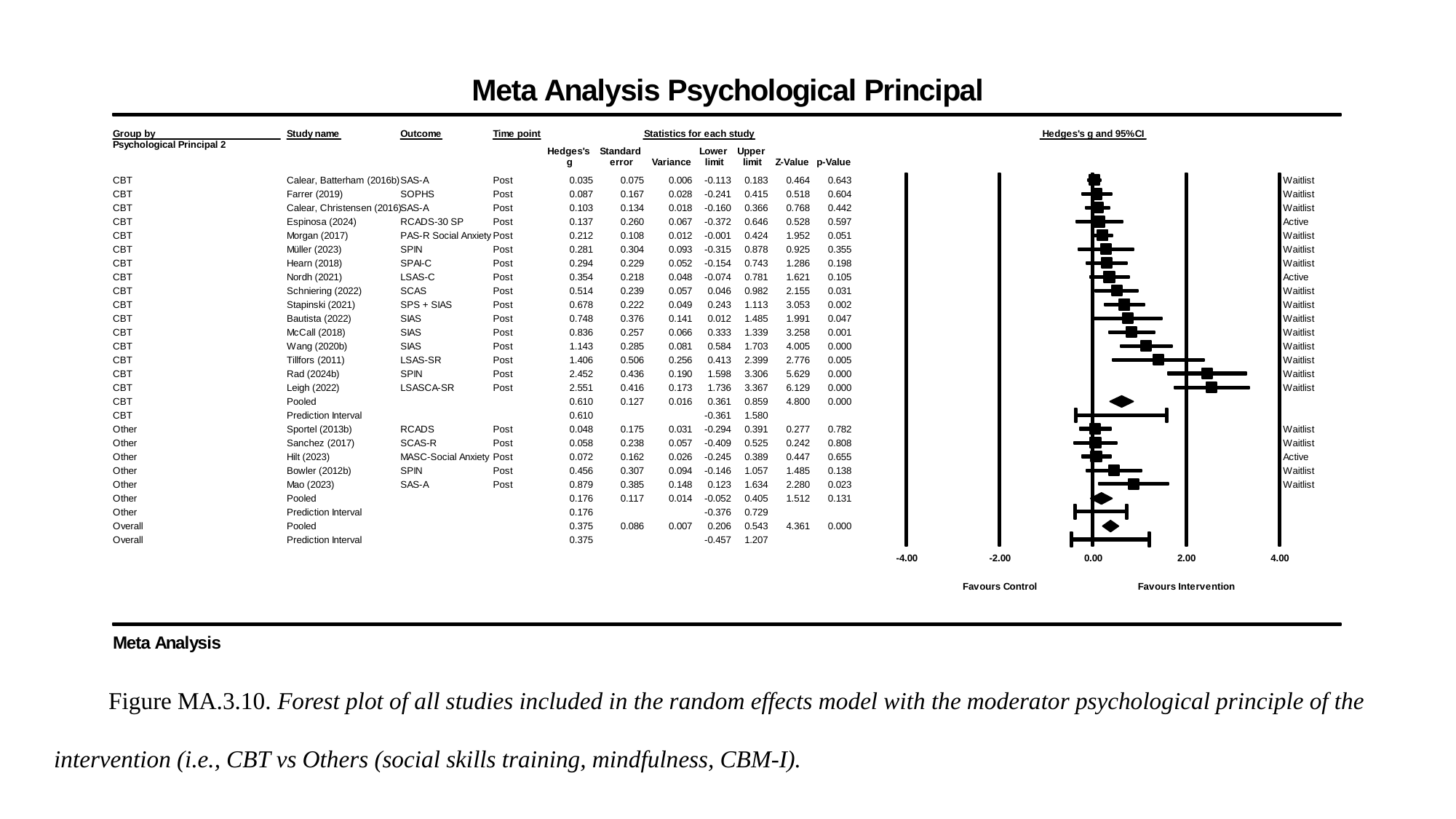

Figure MA.3.10. Forest plot of all studies included in the random effects model with the moderator psychological principle of the intervention (i.e., CBT vs Others (social skills training, mindfulness, CBM-I).

## Slide 12
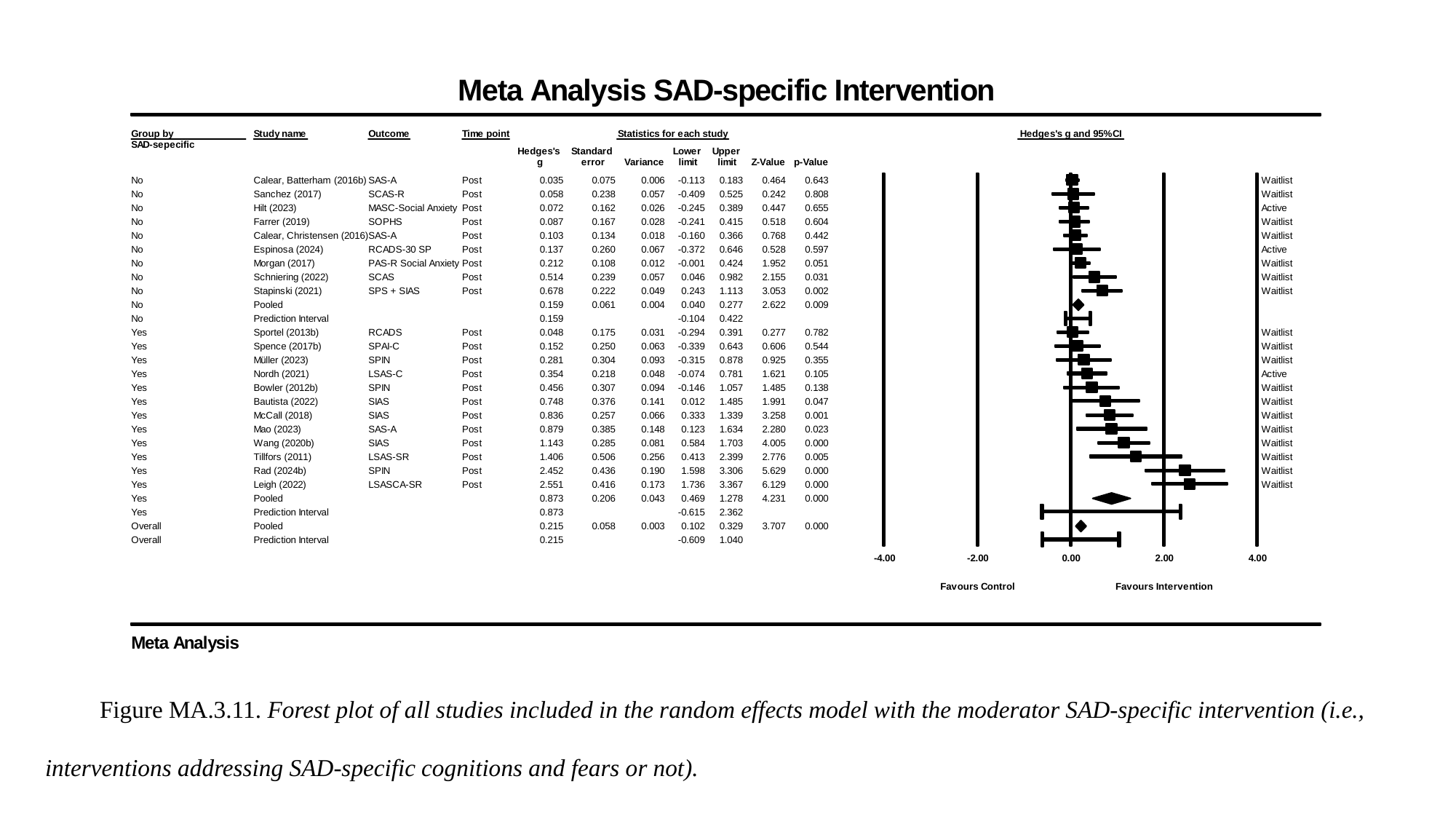

Figure MA.3.11. Forest plot of all studies included in the random effects model with the moderator SAD-specific intervention (i.e., interventions addressing SAD-specific cognitions and fears or not).

## Slide 13
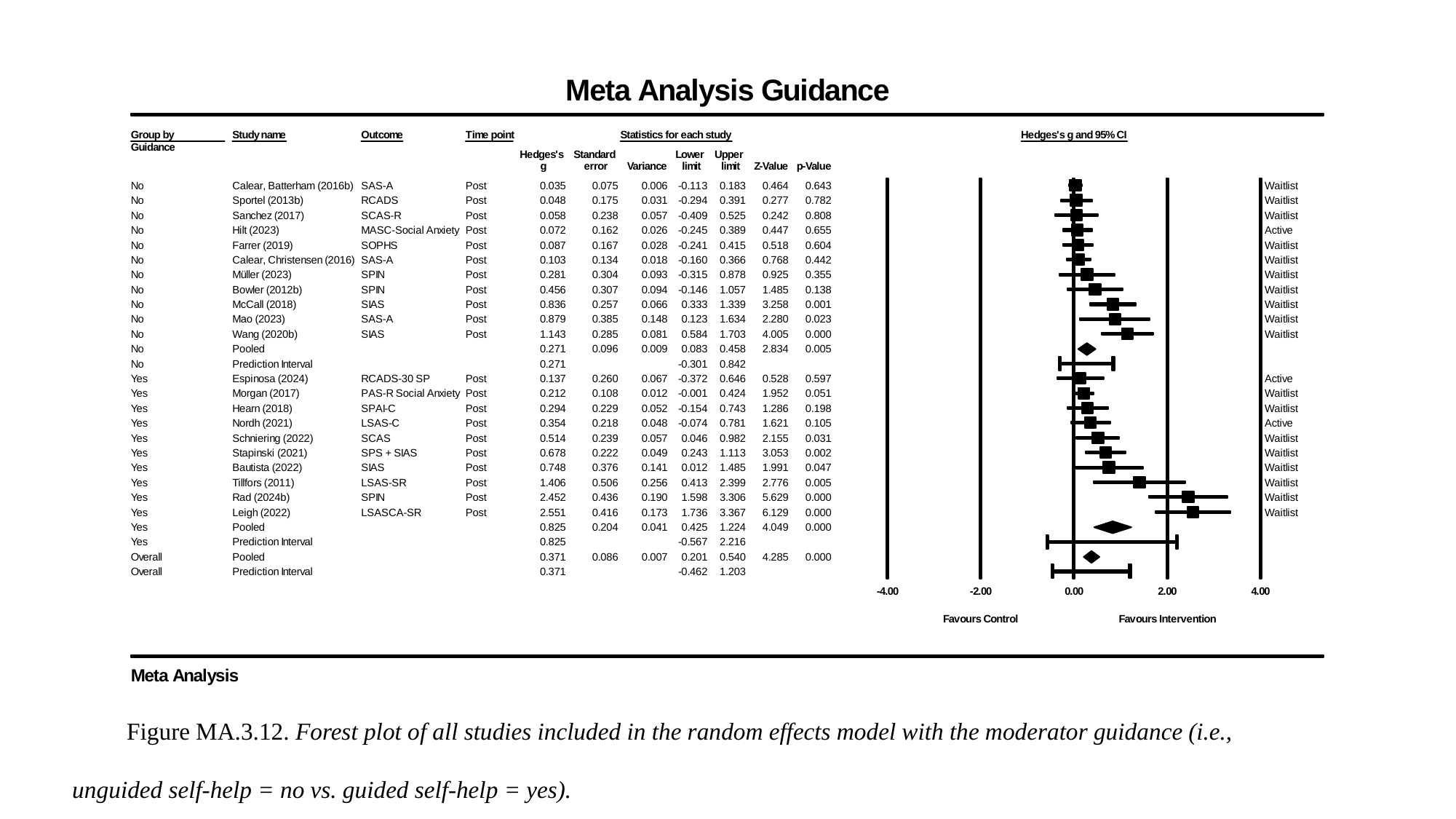

Figure MA.3.12. Forest plot of all studies included in the random effects model with the moderator guidance (i.e., unguided self-help = no vs. guided self-help = yes).

## Slide 14
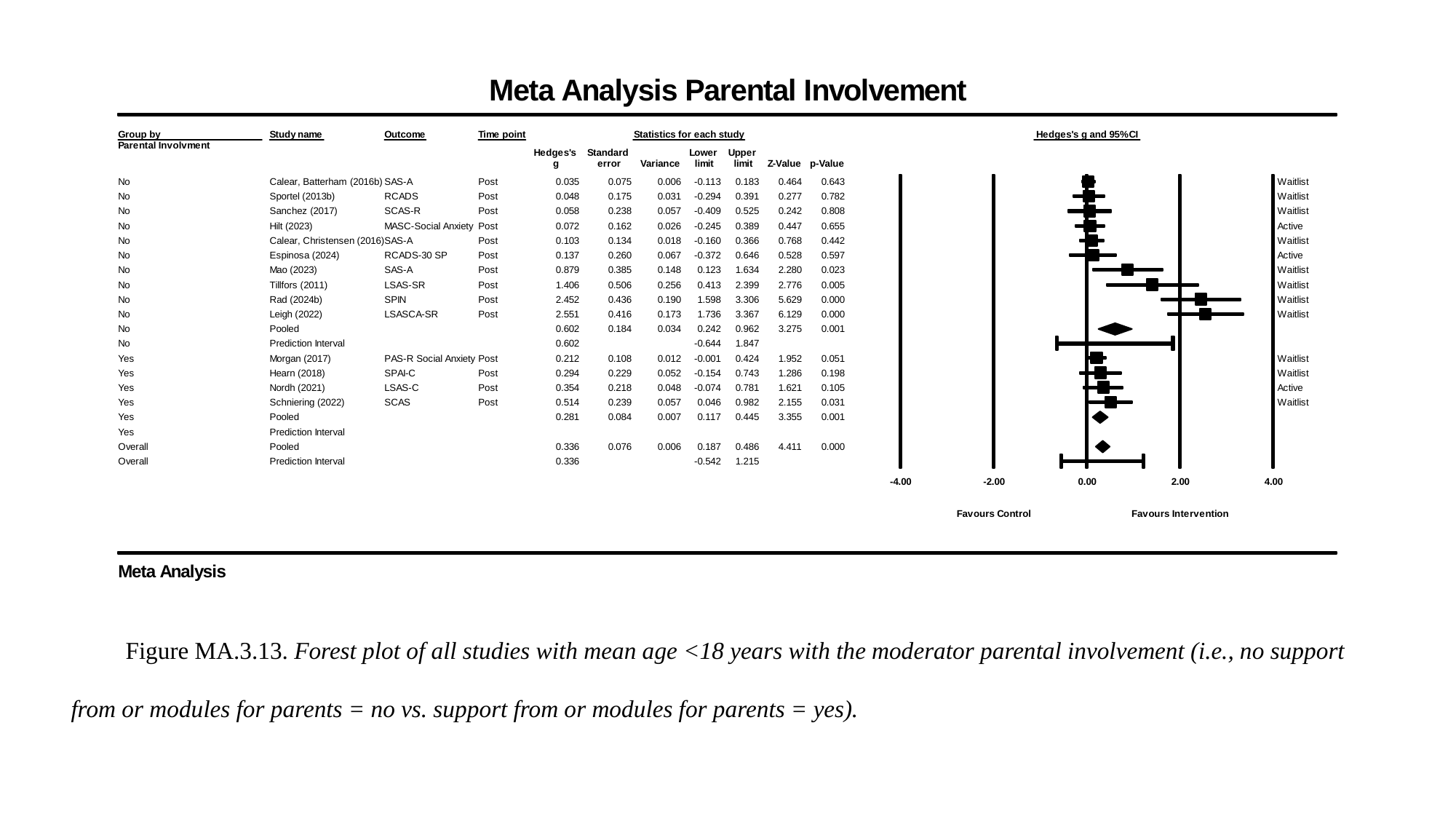

Figure MA.3.13. Forest plot of all studies with mean age <18 years with the moderator parental involvement (i.e., no support from or modules for parents = no vs. support from or modules for parents = yes).
